# Antipsychotic Drug Cariprazine Induces Distinct Cell Death Mechanisms in HeLa and HCT116 Cells as a Potential Inhibitor of Qi-Site of Cytochrome bc1 Reductase

**DOI:** 10.3390/biomedicines14020315

**Published:** 2026-01-30

**Authors:** Marina Mitrovic, Bojana Simovic Markovic, Gvozden Rosic, Marija Ristic, Nemanja Jovicic, Vladimir Jurisic, Jovan Milosavljevic, Sanja Matic, Biljana Ljujic, Dragica Selakovic

**Affiliations:** 1 Department of Medical Biochemistry, Faculty of Medical Sciences, University of Kragujevac, 34000 Kragujevac, Serbia; 2 Center for Molecular Medicine and Stem Cell Research, Faculty of Medical Sciences, University of Kragujevac, 34000 Kragujevac, Serbia; 3 Department of Physiology, Faculty of Medical Sciences, University of Kragujevac, 34000 Kragujevac, Serbia; grosic@fmn.kg.ac.rs (G.R.); jovan.milosavljevic1997@gmail.com (J.M.); dragica984@gmail.com (D.S.); 4 Department of Chemistry, Faculty of Science, University of Kragujevac, 34000 Kragujevac, Serbia; marija.jeremic@pmf.kg.ac.rs; 5 Department of Histology and Embryology, Faculty of Medical Sciences, University of Kragujevac, 34000 Kragujevac, Serbia; nemanjajovicic.kg@gmail.com; 6 Department of Pathophysiology, Faculty of Medical Sciences, University of Kragujevac, 34000 Kragujevac, Serbia; 7 Department of Pharmacy, Faculty of Medical Sciences, University of Kragujevac, 34000 Kragujevac, Serbia; sanjad.matic@gmail.com; 8 Department of Genetics, Faculty of Medical Sciences, University of Kragujevac, 34000 Kragujevac, Serbia; bljujic74@gmail.com

**Keywords:** repurposing, cariprazine, atypical antipsychotic, anticancer, apoptosis, cytochrome bc1 reductase inhibitor, complex III, molecular docking

## Abstract

**Background/Objectives**: Cariprazine (CAR), an atypical antipsychotic drug, exhibits potent anticancer activity; however, its mechanism of action remains unclear. **Methods**: We conducted a comparison of CAR-induced cell death mechanism in HeLa and HCT116 cancer cells and explored its potential role as a Qi-site inhibitor of cytochrome bc1 reductase (complex III). **Results**: CAR induced a dose-dependent cytotoxic effect and triggered apoptosis in both cell lines; however, the mitochondrial responses were distinctively different. HeLa cells exhibited significant mitochondrial membrane depolarization, significant cytochrome c release, a strong increase in the Bax/Bcl-2 ratio, elevated caspase-3 activation, and notable S phase arrest along with autophagy induction, indicating that mitochondria-driven apoptosis occurred rapidly. In contrast, HCT116 cells showed moderate mitochondrial dysfunction, moderate cytochrome c release, enhanced suppression of Akt signaling, and significant G0/G1 phase arrest, which are consistent with a slower and mixed apoptotic response. The findings from molecular docking studies predicted that CAR had stable binding at the Qi site and showed interactions at the Qi site that were comparable to those of antimycin A, thereby suggesting its possible inhibitory effect on complex III. **Conclusions**: The results from our study indicate the engagement of CAR-activated apoptotic pathways that are specific to different types of cancer cells, and hence suggest that CAR may act as a new anticancer drug by potentially directing its action towards the mitochondrial Qi-sites of complex III.

## 1. Introduction

Drug repurposing has become a highly effective strategy for accelerating the discovery of anticancer agents, particularly among neuropsychiatric drugs that exhibit a range of biochemical activities beyond their central nervous system targets [1,2,3].

Epidemiological studies have shown that people with schizophrenia are less likely to suffer from cancer, which indicates the ability of various antipsychotics to inhibit the proliferation of cancer cells [4,5,6,7,8,9]. Furthermore, several antipsychotics can decrease the expression levels of the ABCG2 protein, which could help resensitize resistant cancer cells to chemotherapeutics, as seen with cariprazine [10]. Therefore, these findings have raised an interest among scientists in exploring whether clinically approved antipsychotic medications could demonstrate selective toxicity towards cancer cells through mechanisms that are distinct from their intended neuropsychiatric purposes.

Cariprazine (CAR) is an atypical antipsychotic that has high affinity as a partial agonist for both D_2_/D_3_ and 5-HT_1_A receptors while acting as an antagonist at 5-HT_2_A receptors. It is widely used as an approved treatment for schizophrenia and bipolar I disorder [11], and it serves as an adjunctive therapy to antidepressants for major depressive disorder [12]. The potential of CAR as an anticancer agent has not been sufficiently examined, despite the fact that it demonstrates a wide spectrum of pharmacodynamic effects. Initial studies suggest that various antipsychotics with structural similarities to CAR, such as aripiprazole and brexpiprazole, can inhibit mitochondrial function, increase oxidative stress, or induce apoptosis in cancer models. This indicates that CAR may have comparable or potentially enhanced bioactivities [13,14,15]. Moreover, our previous study has established that CAR exhibited strong anticancer effects on triple-negative breast cancer (TNBC) cells (both in vitro and in vivo TNBC models), which indicated that its antitumor effects may be more extensive than its therapeutic effects for neuropsychiatric disorders. However, the exact mechanisms behind these effects have not been clearly defined [16].

One promising new therapeutic approach in oncology is targeting mitochondria with anticancer drugs. These drugs can disrupt electron transport chain (ETC) complexes, which are responsible for cellular metabolism regulation, apoptotic signal integration, and the generation of ROS in mitochondria to inhibit tumor progression [17,18,19]. Among all ETC complexes, cytochrome bc1 reductase (complex III) plays a crucial role in the Q-cycle [20] and this process has been identified as essential for the development of many tumors [21]. The Qi site, located in the cytochrome b subunit of complex III, binds ubiquinone and several small-molecule inhibitors, including antimycin A [22,23,24,25,26]. Qi-site inhibitors are known to increase the generation of ROS, decrease the mitochondrial membrane potential (ΔΨm), and cause intrinsic apoptosis. In cancer cells, this process includes the release of cytochrome c, the activation of caspases, and the modification of autophagy [27,28,29]. CAR, an atypical antipsychotic with high lipophilicity, possesses physicochemical characteristics that facilitate mitochondrial retention, and several atypical antipsychotics with similar structures, including aripiprazole, risperidone, and clozapine, have been demonstrated to impair mitochondrial respiration by inhibiting various electron transport chain (ETC) complexes, including complex III [30]. Moreover, various psychotropic drugs have been shown to inhibit ECT and induce oxidative stress in cancer cells [31]. Indeed, it has been demonstrated that cariprazine, aripiprazole, and brexpiprazole effectively inhibit respiratory complex I via its ubiquinone-binding channel [32].

Many structural studies have identified conserved Qi-site residues among mammalian cytochrome b structures, such as Asp228, His201, Ser35, Trp31, Lys 227, Ser 205 and Phe220, which are essential for the binding of ubiquinone or inhibitors like antimycin A [33,34]. Such conservation expands the potential for drug-like compounds to bind to the Qi pocket. PASS predictions in our preliminary computational screening revealed that CAR may potentially inhibit the Qi-site of cytochrome bc1 reductase, with a probability greater than several of its recognized psychotropic actions. Also, similar to UQ and antimycin A, CAR had favorable Qi pocket binding energies and interaction patterns in structural molecular docking. These results led to the hypothesis that CAR may act as potential anticancer agent by targeting complex III at the Qi site. However, the cellular effects of Qi-site blocking in different types of tumors can vary according to factors like mitochondrial dependency, survival signals, and metabolic flexibility. HeLa (cervical cancer) and HCT116 (colorectal cancer) cells are characterized by entirely different glycolytic dependency, mitochondrial sensitivity, and activation of the Akt pathway [35,36,37]. These differences make them attractive cancer cell types to investigate the cellular mechanism through which the potential interference of CAR with the Qi-site activates apoptosis, autophagy, and cell-cycle arrest. Therefore, the aim of this study is to explore the anticancer effects of CAR in HeLa and HCT116 cells, as well as to assess whether its mechanisms of inducing cell death, such as mitochondrial dysfunction, intrinsic apoptosis, autophagy induction, and cell-cycle arrest, align with its predicted role as a Qi-site inhibitor of cytochrome bc1 reductase. Thus, we present, for the first time, a comprehensive mechanistic framework linking CAR’s anticancer activity to its potential inhibition of the Qi-site of mitochondrial complex III by integrating in vitro cytotoxicity assays, apoptosis and autophagy profiling, cell-cycle analysis, and in silico docking.

## 2. Materials and Method

### 2.1. Cell Lines and Reagents

Cell lines from the American Type Culture Collection were used: MRC-5, a non-cancerous human fibroblast lung cell line (ATCC-CCL-171); HeLa—cervical (CRM-CCL-2-ATCC) and HCT116—colon (CCL-247-ATCC)—human cancer cell lines, cultured in Dulbecco’s Modified Eagle’s Medium (catalog number D5671—Sigma-Aldrich, St. Louis, MO, USA), supplemented with fetal bovine serum (10%—catalog number F7524—Sigma-Aldrich), streptomycin (100 μg/mL—catalog number 11074440001-Roche, Sigma-Aldrich, St. Louis, MO, USA), and non-essential amino acids (1%—catalog number M7145). Cultures were incubated at 37 °C in a 5% CO_2_ humidified environment. Merck Millipore, Distributor NOVOS D.O.O, Belgrade, Serbia (CAS No. 51-21-8) supplied 5-fluorouracil (5-FU). The Department of Psychiatry at the University Clinical Center Kragujevac supplied antipsychotic drugs such as Reagila (cariprazine-CAR), Aripiprazole, and Eglonyl (Sulpiride-SPD). Dr. Marija S. Ristić (Faculty of Science, Department of Chemistry, University of Kragujevac) generously contributed the pure antipsychotic chemical CAR (Gedeon Richter, Budapest, Hungary). All chemicals studied were dissolved in DMSO to a final concentration of 10 mM (Sigma-Aldrich, St. Louis, MO, USA) (total DMSO concentration of 0.5%). The concentrations of all tested antipsychotics and 5-FU were 5, 10, 20, 30, 40, 50, and 100 μM.

### 2.2. Cytotoxicity Assessment—MTT Assay

The cytotoxicity of three drugs (Reagila, Aripiprazole, and Eglonyl), pure CAR, and 5-FU (5, 10, 20, 30, 40, 50, and 100 μM) was examined in HCT116 and HeLa cancer cells and the MRC-5 cell line. The MTT assay quantifies cell metabolism by analyzing the transformation of yellow MTT to purple formazan. Cells, plated at 3000 per well in 96-well plates using DMEM medium with 10% FBS, 100 U/mL penicillin, and 100 μg/mL streptomycin, were incubated for 24 h at 37 °C and 5% CO_2_. Following treatment with various antipsychotic drug doses and 5-FU, cells were further incubated for 24, 48, and 72 h. After adding a 1:10 MTT reagent for two hours at 37 °C in a 5% CO_2_ incubator, DMSO was added to dissolve the purple precipitate. Optical density at 595 nm was measured with a microplate reader (Zenyth 3100, Anthos Labtec Instruments, Salzburg, Austria). The MTT test measures the absorbance of purple formazan crystals to determine cell viability and cytotoxicity. Percentage of cell viability was calculated using the following formula: Absorbance of treated cells/Absorbance of control cells × 100%. % of cytotoxicity was calculated using the formula: % of cytotoxicity = (1 − Absorbance of treated cells/Absorbance of control cells) × 100%. The Quest GraphTM IC_50_ Calculator, developed by AAT Bioquest, Inc., Pleasanton, California, USA. and based on a four-parameter logistic regression model, was used to determine the IC50. AAT Bioquest, Inc.’s website provides detailed information on this calculation tool (https://www.aatbio.com/tools/ic50-calculator, accessed on 17 January 2026). All IC_50_ values are reported as mean ± SEM, calculated from at least three independent biological experiments (*n* = 3), each performed with technical triplicates. In accordance with standard procedure in similar in vitro cytotoxicity studies, SEM was employed rather than confidence intervals. To find the tumor-selectivity index (SI), we divide the mean IC_50_ in tumor cells by the mean IC_50_ in non-cancerous cells.

### 2.3. Flow Cytometric Assessment of Apoptosis

The apoptosis and necrosis in HeLa and HCT116 cells treated with CAR and 5-FU at respective IC_50_ doses were examined by flow cytometry. Cells that have been treated were labeled with Annexin-V-FITC/7-AAD (STEM CELL Tech, Vancouver, BC, Canada; Catalog #100-0339). First, the cells were centrifuged at 400× *g* for 5 min at RT, washed with PBS, and then resuspended in 1× Annexin-V Binding Buffer. Incubation was performed on the cells with 10 μL of 7-AAD and 5 μL of Annexin-V-FITC. Following a vortexing step, the samples were incubated in the dark at RT for 15 min, further centrifuged, and resuspended in 300 μL of 1× Annexin-V Binding Buffer. A Cytomics FC500 flow cytometer, made by Beckman Coulter, Brea, CA, USA, was used to evaluate the labeled cells.

### 2.4. Assessment of Apoptosis Using Dual Staining with AO/EtBr

Apoptosis was evaluated using dual-stain Acridine Orange (AO) and Ethidium Bromide (EtBr) techniques, with fluorescence microscopy allowing for differentiation of viable, non-viable, and apoptotic cells. EtBr specifically labels dead cells, producing red fluorescence upon DNA binding; conversely, AO stains both live cells (green fluorescence) and dead cells (orange-red fluorescence). The experimental procedure included seeding HCT116 and HeLa cell lines at a density of 20,000 cells per well in 24-well culture plates and incubated at 37 °C with 5% CO_2_ for 24 h, treated with 5-FU and CAR (IC_50_) for 48 h, and then stained with AO (2.5 μg/mL) and EtBr (2.5 μg/mL) (Sigma, St. Louis, MO, USA) for 20 min. The FLUO500T Trinocular inverted fluorescent microscope (Gramma Libero, Belgrade, Serbia) was used to investigate the morphology of healthy, apoptotic, and dead cells. The 2020 S-EYE Setup Microscope camera (Shenzhen Hayear Electronics Co., Ltd., Shenzhen, China) and S-EYE_Setup-1.6.0.11 software were employed to take all the images. Then, using ImageJ software, version ij153-win-java8 all the images were processed and analyzed, and their scales were adjusted to 100 μm or 40× magnification. The mean intensity of fluorescence at 525 nm and 590 nm was measured.

### 2.5. Flow Cytometric Analysis of Protein Expression

The cells were placed into 24-well plates with 10 × 10^4^ cells/well in complete DMEM medium. After 24 h of incubation at 37 °C with 5% CO_2_, they were treated with CAR (IC_50_) for 48 h. After collection, the cells were then fixed in 4% paraformaldehyde for 30 min at room temperature. Later, they were permeabilized with 0.2% Tween 20 in PBS, and finally, they were blocked for 30 min with 0.1% Tween in PBS. The cells were subsequently labeled with primary antibodies at a concentration of 1:100. These antibodies included a mouse antibody against human active caspase-3 (# 9661, Cell Signaling, Danvers, MA, USA), a mouse antibody against human Bax (Santa Cruz Biotech, Inc., Dallas, TX, USA, N20, # sc-493), a mouse antibody against human Bcl-2 (Santa Cruz Biotech, Inc., Dallas, TX, USA, DC21, #sc-783), and a rabbit antibody against human phospho-Akt (Ser-473) (#9271, Cell Signaling, Danvers, MA, USA). Subsequently, the cells were exposed to secondary antibodies, which included goat anti-mouse FITC (1:200), anti-rabbit Alexa Fluor 488 (#4412, Cell Signaling, Danvers, MA, USA) (1:200), and rabbit anti-human SQST1M/p62-Alexa Fluor 488. To further assess the labeled cells, a Cytomics FC500 flow cytometer (Beckman Coulter, 205 USA) was employed.

### 2.6. Assessing the Potential of the Mitochondrial Membrane (ΔΨM) Using the JC-10 Method

The JC-10 dye was used to evaluate ΔΨM. Live cells retain JC-10 in mitochondria, where it forms red fluorescence aggregates. However, JC-10 fluoresces necrotic and apoptotic cells green and stains the cytoplasm monomeric. HCT16 and HeLa cells were plated in 24-well plates at a density of 2 × 10^4^ cells per well, incubated at 37 °C with 5% CO_2_ for 24 h, treated with CAR (IC_50_) for 48 h and then stained for 20 min with 2.5 μM JC-10 dye (Enzo Life Sciences, Farmingdale, NY, USA) in PBS at 37 °C and 5% CO_2_. The Gramma Libero FLUO500T trinocular inverted fluorescent microscope was used to detect JC-10 absorption in cells. We employed the 525/590 nm fluorescent emission ratio for quantitation. Images were captured using the 2020 S-EYE Setup Microscope camera and the S-EYE_Setup-1.6.0.11 software. Analysis of images was conducted using ImageJ. To measure ΔΨM using flow cytometry following CAR treatment, the cells were pelleted and left to incubate in warm PBS with 2.5 μM JC-10 dye for 15–30 min at room temperature in the dark. Then, the label cells were evaluated with a Cytomics FC500 flow cytometer from Beckman Coulter USA. The data were visualized as bar histograms after analysis using the FlowJo program, version 10.10.

### 2.7. Immunofluorescence Analysis of Protein Expressions

HeLa and HCT116 cells were cultured in 24-well plates and exposed to CAR (IC_50_) for a period of 48 h. They were fixed with 4% paraformaldehyde for 30 min at RT, permeabilized with 0.2% Tween 20 in PBS, and blocked with 0.1% Tween 20. After a one-hour incubation period at room temperature, a rabbit anti-human phospho-Akt (Ser-473) antibody, a human active-caspase-3 antibody, and human cytochrome c antibody (G7421, Promega, Madison, WI, USA, 1:100) were added. Subsequently, a goat anti-mouse FITC (1:200) or anti-rabbit Alexa Fluor 488 (1:200) (Cell Signaling, 4412) was added to the samples for 1 h. After that, they were counterstained with PI and examined under an inverted FLUO500T Trinocular fluorescent microscope (Gramma Libero). Images were examined at a magnification of 40× at a scale of 100 μm using ImageJ software. Mean fluorescence intensity (MFI) was measured independently within cytosolic (diffuse) and mitochondrial (punctate) regions of interest (ROIs) utilizing ImageJ software, based on three separate images from three different experiments. The mean ratio (cytosolic cyt c MFI/mitochondrial cyt c MFI) was used as an index of cytochrome c redistribution from mitochondria to cytosol.

### 2.8. Cell Cycle Analysis

CAR-treated HCT116 and HeLa cells were collected, centrifuged, and washed in PBS. Cold ethanol was then diluted dropwise to a final concentration of 70%. The cells were fixed on ice for a minimum of 4 h, then rinsed in PBS and transferred to staining buffer (PBS with 50 μg/mL propidium iodide and 100 μg/mL RNase A) at RT for 15 to 30 min in the dark. The histogram bars show the percentages of cells dispersed to different cell cycle phases, which were determined using the FlowJo software tool.

### 2.9. Evaluation of Reactive Oxygen Species (ROS)

The generation of ROS was assessed through the dichloro fluorescein diacetate assay (DCFDA) (Abcam, Waltham, MA, USA). In summary, Hela and HCT116 cells were exposed to CAR (IC_50_) over 48 h, and then were washed, trypsinized, and incubated with DCFDA (20 μM) for 30 min in the dark at 37 °C and 5% CO_2_. An FC500 flow cytometer from Cytomics (Beckman Coulter USA) was used to measure the signals of DCFDA fluorescence distribution. The results are presented as the mean fluorescence intensity (MFI) of DCFDA compared to the control.

### 2.10. Predicting Biological Activity of CAR Using PASS and MetaPASS Tools

PASS (Predicted Activity Spectrum for Substances) online server (https://way2drug.com/PassOnline/predict.php, accessed on 16 October 2025) was applied to predict the biological activity of CAR, applying bioinformatics to examine chemical structures for potential biological, pharmacological, and toxicological effects [38]. The prediction is based on a structure–activity relationship (SAR) study of more than 250,000 biologically active compounds, each of which has more than 3500 types of biological activities. PASS can achieve high predictive performance for certain activity classes; however, predictive accuracy (in some cases up to 95%) varies across biological targets and should not be interpreted as universally applicable. Accordingly, PASS outputs were used in this study as hypothesis-generating indicators to predict potential mechanisms of action rather than as definitive evidence of biological activity [39]. The results give a list of activities in order of the difference between the probabilities of being active (Pa) and inactive (Pi). A greater positive difference indicates a higher predicted level of biological activity. The software generates these predictions and categorizes activity levels according to specific Pa criteria (Pa > 0.7 indicating strong activity, 0.5 < Pa ≤ 0.7 indicating moderate activity, and Pa < 0.3 indicating low activity) based on the CAR chemical structure provided from PubChem (https://pubchem.ncbi.nlm.nih.gov/#query=cariprazine, accessed on 10 October 2025). Additionally, the MetaPASS program (https://way2drug.com/MetaPASS/, accessed on 16 October 2025) was used to evaluate the biological activity spectrum of organic compounds utilizing CAR as a ligand to find related molecules, considering their metabolic pathways.

### 2.11. In Silico Study of CAR with Cytochrome bc1 Reductase Using Advanced Molecular Docking Tools

A molecular docking study was performed using SwissDock 2024 (https://www.swissdock.ch/, accessed on 18 October 2025) [40], which employs either the Attracting Cavities 2.0 [41] or AutoDock Vina 1.2.5 method [42]. AutoDock Vina is known for being precise and fast, while Attracting Cavities makes more reliable predictions but takes longer to calculate.

The ligand CAR was prepared using SMILES notation from PubChem [43] and parameterized with SwissParam, utilizing an MMFF-based approach compatible with the CHARMM36 force field [44]. The 3D structures of cytochrome bc1 reductase were obtained from the PDB database (1NTK) (https://www.rcsb.org/structure/1NTK, accessed on 18 October 2025), and (1NTZ) (https://www.rcsb.org/structure/1NTZ, accessed on 18 October 2025), with essential preparations such as protonation at physiological pH and the incorporation of missing side chains and hydrogen atoms. The Attracting Cavities (AC) docking protocol was used as the primary method, as it is optimized for ligand placement within predefined binding pockets and does not rely on user-defined exhaustiveness parameters. The docking search space was restricted to the experimentally validated Qi binding pocket of cytochrome bc1 reductase, based on crystal structures complexed with ubiquinone (PDB ID: 1NTZ) and antimycin A (PDB ID: 1NTK). Default SwissDock sampling settings were applied, ensuring exhaustive exploration of ligand conformations within the selected cavity. In order to validate the uniformity of the poses and the predicted binding energies, AutoDock Vina was used as a secondary method. The docking scores were calculated using two scoring functions: the AC docking score and the SwissParam score, which enabled the ranking of ligand poses based on their binding energy (Kcal/mol). Post-docking analysis utilized UCSF Chimera (https://www.rbvi.ucsf.edu/chimera/, accessed on 2 November 2025) and BIOVIA Discovery Studio (https://discover.3ds.com/discovery-studio-visualizer-download, accessed on 2 November 2025) to visualize binding interactions, identifying key interactions like π-π stacking, hydrophobic contacts, and hydrogen bonds. Comprehensive 2D interaction diagrams show the residues involved in binding, emphasizing the stability and specificity of the CAR-Qi of cytochrome bc1 complexes. Superimposition of the three-dimensional structures of complexes was performed by PyMOL 3.1.6.1 software (https://www.schrodinger.com/platform/products/pymol, accessed on 10 November 2025).

### 2.12. Statistical Analysis

The following statistical parameters were calculated: mean value and standard error of the mean as basic descriptive statistics. The normality of distribution parameters was checked by the Kolmogorov–Smirnov test. A two-tailed Student’s *t*-test or nonparametric Mann–Whitney rank-sum test, depending on the normal distribution, was performed. Result processing was carried out using the SPSS 26.0 software package (SPSS Inc., Chicago, IL, USA). A *p*-value below 0.05 was considered to be significant.

## 3. Results

### 3.1. Aripiprazole, Sulpiride (Eglonyl), and Cariprazine (Reagila)’s Cytotoxic Effects in Cervical and Colon Cancer Cells

**Figure 1:**
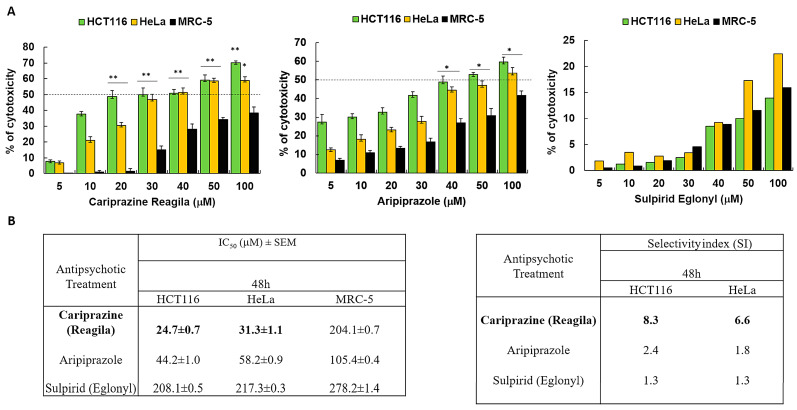
Antipsychotic drugs reduce the viability of HCT116 and HeLa cancer cells. (**A**) Three different antipsychotic drugs, Reagila (cariprazine), Aripiprazole, and Eglonyl (sulpiride), were administered at varying concentrations (5, 10, 20, 30, 40, 50, and 100 µM) for a duration of 48 h to HCT116, HeLa, and MRC-5 cells. The MTT assay was used to measure the % of cell viability. The dotted line indicates drug’s half maximum inhibitory concentration (IC_50_). The bar graphs illustrate the mean percentage of cell viability ± SEM (*n* = 3). * *p* < 0.05 and ** *p* < 0.01 indicate statistical significance compared to the control group. (**B**) The tables present the selectivity index (SI) of the drugs after 48 h of treatment of HCT116 and HeLa cells, along with the mean IC_50_ (µM) values ± SEM for the tested drugs on HCT116, HeLa, and MRC-5 cells. Cariprizine (bold) was the most cytotoxic to cancerous cells compared to other antipsychotics.

We investigated the cytotoxic effects of three psychotropic drugs: Reagila (cariprazine), Aripiprazole, and Eglonyl (sulpiride) on HCT116 colon cancer cells, HeLa cervical cancer cells, and non-cancerous human fibroblast lung cell line MRC-5. The results showed that both aripiprazole and CAR caused a significant decrease in cell viability in both cancer cell lines in a dose-dependent manner when compared to MRC-5 cells. Sulpiride (Eglonyl) exhibited a negligible decrease in viability of HCT116 and HeLa cells when compared to MRC-5 cells (Figure 1A). Compared to the other drugs tested, CAR had the highest cytotoxic effect on HCT116 and HeLa cells, with the mean IC_50_ values of 24.0 ± 0.5 μM and 31.5 ± 0.9 μM, respectively. The mean IC_50_ value of CAR against MRC-5 cells was 204.1 ± 0.7 μM, suggesting non-significant cytotoxicity in these cells. Sulpiride’s IC_50_ values exceeded 200 μM for both cancer cells and MRC-5 cells, suggesting that sulpiride lacks selectivity as an antitumor agent for the treatment of colon and cervical cancers. CAR demonstrated a selectivity index (SI) of 8.5 for HCT116 cells and 6.5 for HeLa cells 48 h post-treatment. This finding indicates a preferential CAR’s cytotoxic effect on cancerous cells in comparison to the non-cancerous MRC-5 cells (Figure 1B).

### 3.2. The Cytotoxicity of the Pure Chemical CAR on HeLa Cells and HCT116 Cells Is Dose- and Time-Dependent

**Figure 2:**
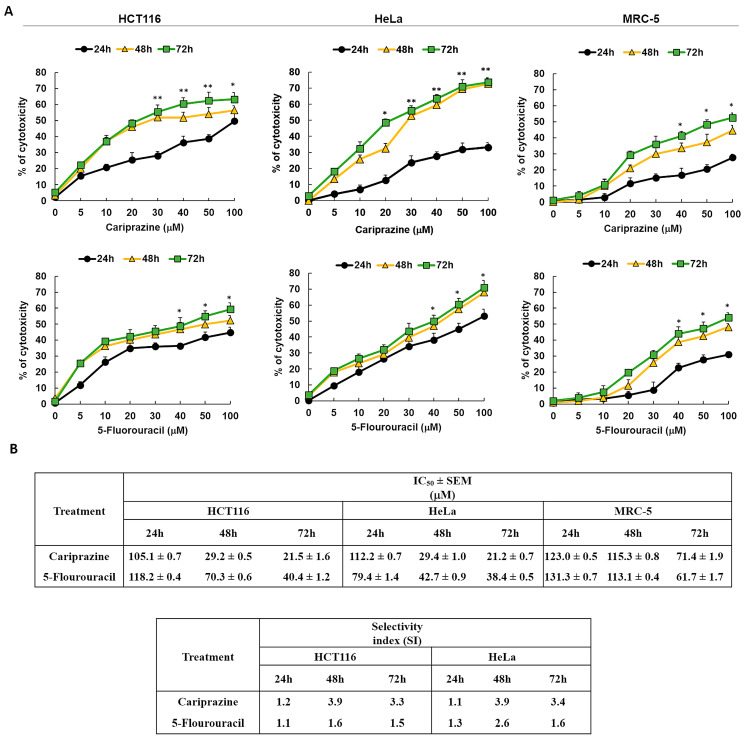
The cytotoxicity of pure chemical CAR and 5-FU on HeLa, MRC-5, and HCT116 cells depends on both dosage and time. (**A**) An MTT test was used to assess the cytotoxicity of CAR and 5-FU following treatment of cells for 24, 48, and 72 h at doses of 5, 10, 20, 30, 40, 50, and 100 mM. The % of cytotoxicity was plotted against the concentrations of 5-FU and CAR to produce the dosage and time response curves. Dot points show the mean cytotoxicity % ± SEM (*n* = 3). * *p* < 0.05 and ** *p* < 0.01 present the significance levels. (**B**) The tables present the selectivity index (SI) and the mean IC_50_ values ± SEM and for CAR and 5-FU in HCT116, HeLa, and MRC-5 cells after treatment durations of 24, 48, and 72 h.

Next, we examined the cytotoxic effects of pure chemical CAR and 5-FU, a reference anticancer agent, on HCT116, HeLa, and MRC-5 cells to validate the cytotoxicity screening results of the pharmaceutical CAR medication. The results showed that the cytotoxic effects of CAR and 5-FU were dose- and exposure-time-dependent (Figure 2A). CAR showed significant cytotoxicity in HCT116 and HeLa cells compared to MRC-5 cells, based on its IC_50_ and SI values at all time intervals. The IC_50_ and SI values of CAR showed a constant cytotoxicity profile over all time points tested for the HCT116 and HeLa cells. In Figure 2B, the comparison with 5-FU reveals CAR’s much higher SI, suggesting that it is a more potent antitumor agent with respect to the tested cell lines in both colon and cervical cancers.

### 3.3. CAR Induces Apoptosis in HCT116 and HeLa Cells

**Figure 3:**
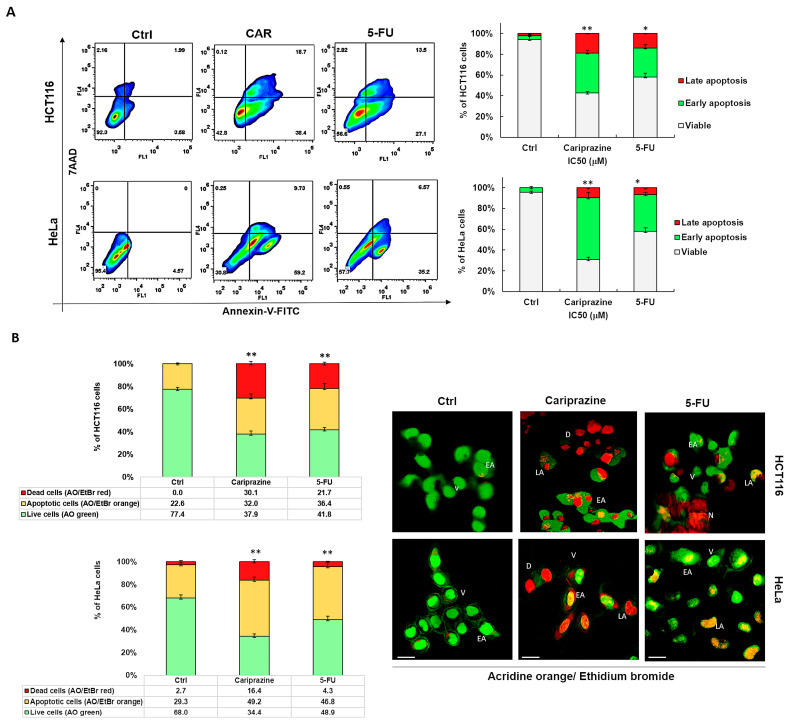
CAR causes HeLa and HCT116 cells to undergo apoptotic cell death. (**A**) Using IC_50_ values of CAR (30 μM) and 5-FU (70.3 μM—HCT116—and 43 μM—HeLa), apoptosis was evaluated in HCT116 and HeLa cells 48 h after treatment. Annexin V-FITC/7AAD was used for flow cytometry and dual labeling. The analysis revealed live cells (lower left quadrant), necrotic cells (upper left quadrant), early apoptotic cells (lower right quadrant), and late apoptotic cells (upper right quadrant). The bar graphs show the mean % of viable, early apoptotic, and late apoptotic cells ± SEM (*n* = 3). Statistically significant differences were indicated by * *p* < 0.05 and ** *p* < 0.01 in comparison to control. (**B**) Early apoptosis is shown by yellow-green nuclei in CAR-treated cells and round and green AO-stained nuclei in untreated cells. Orange or red nuclei stained with EtBr indicate late apoptotic cells. The bar graphs display the mean % of live and apoptotic cells ± SEM (*n* = 3). Levels of significance are shown as ** *p* < 0.01 relative to the control (Ctrl). A 40× magnification and scale bars of 100 μm are used. (V—viable; EA—early apoptotic; LA—late apoptotic; D—dead; N—necrotic).

We next examined the specific type of cell death that CAR induced in HCT116 and HeLa cells after performing the cytotoxicity assays. To evaluate apoptosis and necrosis, cells were stained with Annexin V-FITC/7AAD after a 48 h treatment with IC_50_ concentrations of CAR (30 μM) and 5-FU (70.3 μM—HCT116 and 43 μM—HeLa). The untreated group exhibited a significant majority of viable cancer cells (95.4% of HeLa and 92.3% of HCT116), with only a minor proportion exhibiting early apoptosis (3.58% and 4.57%, respectively). In control HCT116 cells, 2.16% of cells were necrotic and 1.99% were late apoptotic. In untreated HeLa cells, there were no necrotic or late apoptotic cells. CAR successfully triggered apoptosis in both cancer cell types. However, CAR showed the most evident and significant early apoptotic effect in 59.2% of HeLa cells, in contrast to 38.4% in HCT116 cells. The early apoptotic effect of 5-FU was observed in 35.2% of HeLa cells and 27.1% of HCT116 cells. Conversely, CAR induced a significantly greater late apoptotic effect in 18.7% of HCT116 cells, whereas only 9.73% of HeLa cells exhibited this effect (Figure 3A). The Annexin V/7AAD staining of the cells was further supported by AO/EtBr labeling of both CAR-treated and untreated cells, offering additional evidence that CAR may induce apoptosis. Subsequent AO/EtBr labeling of CAR-treated and untreated cancer cells validated Annexin V/7AAD staining, providing further confirmation that CAR may cause apoptotic cell death. The HCT116 and HeLa cell percentages of AO/EtBr-labeled apoptotic cells increased significantly in the cancer cells treated with CAR and 5-FU, with 32.0% and 49.2% for CAR and 36.4% and 46.8% for 5-FU, respectively, when compared to untreated cancer cells. CAR demonstrated a higher percentage of dead cells, with 30.1% observed in HCT116 compared to 16.4% in HeLa cells (Figure 3B).

### 3.4. CAR Modulates Bcl-2 and Bax Expression in Cancer Cells

**Figure 4:**
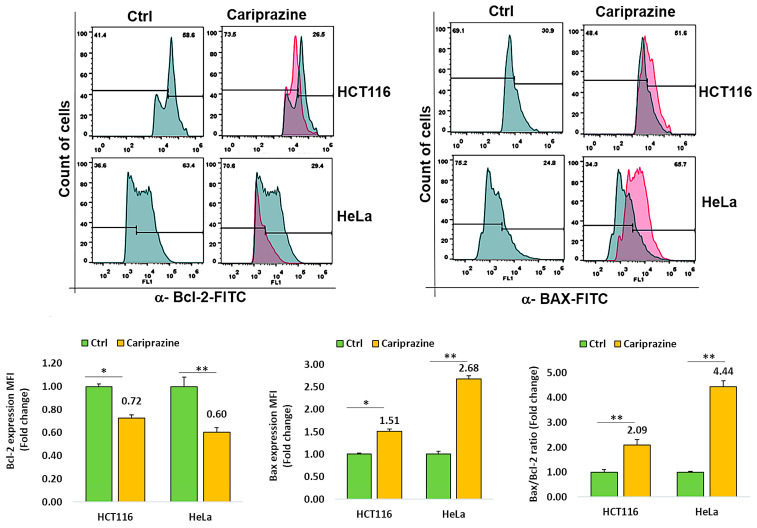
CAR regulates Bcl-2 and Bax protein expression in cancer cells. The expression levels of Bcl-2 and active Bax proteins were determined in untreated and CAR-treated HCT116 and HeLa cells at its IC_50_ value (30 μM), utilizing flow cytometry after a 48 h period. The histogram bars illustrate the mean fold change in protein expression (MFI) ± SEM and the mean fold change in the Bax/Bcl-2 ratio ± SEM (*n* = 3). * *p* < 0.05 and ** *p* < 0.01 indicate significant levels compared to the control group (Ctrl).

To investigate the mechanism through which CAR causes cell death, we tested HCT116 and HeLa cancer cell lines for changes in the expression of Bcl-2 and Bax, two proteins involved in cell death. Cancer cells were incubated with CAR (IC_50_—30 μM) for 48 h, and the protein expression of Bax and Bcl-2 was evaluated via flow cytometric analysis. The obtained results demonstrated a significant elevation in proapoptotic Bax expression, while both cancer cells treated with CAR showed a marked decrease in the protein levels of Bcl-2. In addition, the Bax/Bcl2 ratios in HCT116 and HeLa cells treated with CAR increased by 2.09- and 4.44-fold, respectively, when compared to the untreated cells (Figure 4). CAR-treated HeLa cells exhibited a greater increase in the Bax/Bcl-2 ratio compared to HCT116 cells. These results demonstrate that CAR acts as an efficient regulator of apoptosis in these cancer cells, especially in cervical cancer.

### 3.5. CAR Affects Active Caspase 3 Expression in Colon and Cervical Cancer Cells

**Figure 5:**
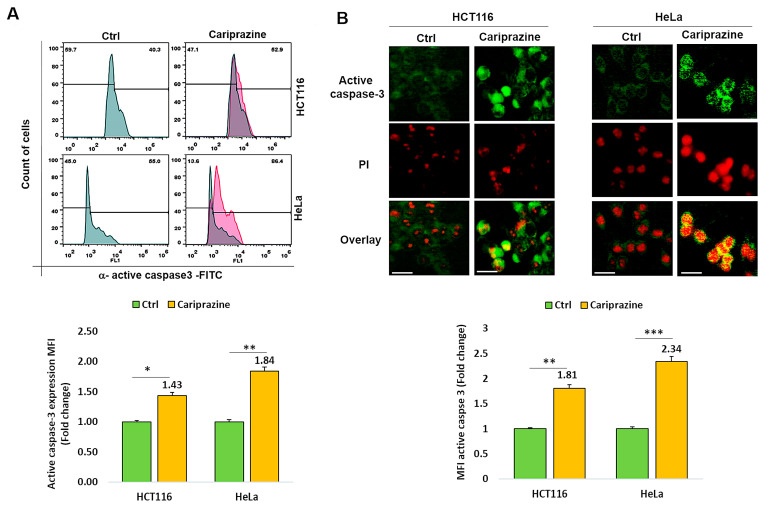
CAR modulates the expression of active caspase 3 in HCT116 and HeLa cells. (**A**) The protein expression (MFI) of active caspase 3 was assessed by flow cytometry in untreated cells and in HCT116 and HeLa cells treated with IC_50_ concentrations of CAR (30 μM) for 48 h. (**B**) The cellular localization and MFI of active caspase 3 were examined using immunofluorescence. An increase in PI red fluorescence in dead cells corresponded with the colocalization of active caspase 3. Histogram bars display the mean fold change in protein expression (MFI) of caspase 3 ± SEM (*n* = 3). Statistical significance was presented by * *p* < 0.05, ** *p* < 0.01, and *** *p* < 0.001, compared to the control group. Scale bars = 100 μm; 40×.

Thereafter, we investigated whether CAR affected cancer cells’ expression of active caspase 3. Figure 5A shows that after 48 h of treatment with CAR (IC_50_—30 μM), active caspase 3 expression was 1.43- and 1.84-fold higher in HCT116 and HeLa cancer cells, respectively, compared to untreated cells. Immunofluorescence evaluations of caspase-3 activation and cellular localization showed that CAR considerably increased the higher expression (MFI) of active caspase-3 in HeLa cells by 2.34-fold, compared to a 1.81-fold increase in HCT116 cells relative to the control (Figure 5B).

### 3.6. CAR Affects Mitochondrial Membrane Potential (ΔΨM) and Promotes Cytochrome c Release

**Figure 6:**
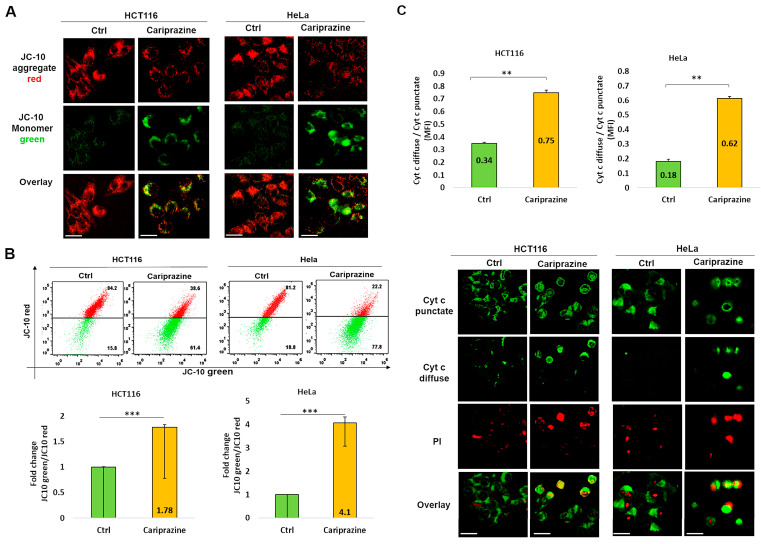
CAR induces changes in mitochondrial membrane potential (ΔΨM) and facilitates the release of cytochrome c in HCT116 and HeLa cells. (**A**) After 48 h of CAR’s treatment (IC_50_—30 μM), HCT116 and HeLa cells were labeled with JC-10; (magnification: 40×, 100 μm scale bar). (**B**) The histogram bars ± SEM show the mean fold change in the JC-10 green/red ratio (*n* = 3). *** *p* < 0.001 vs. control group. (**C**) Immunolocalization of mitochondrial (green punctate) and cytoplasmic (green diffuse) cytochrome c was observed in both control and CAR-treated cancer cells after 48 h. PI red fluorescence increased in dead cells. Bar graphs illustrate the mean ratio of cytosolic (diffuse) to mitochondrial (punctate) mean fluorescence intensity (MFI) of cytochrome c (*n* = 3). The mean ± SEM is shown by the bars. A significant difference (** *p* < 0.01). 40× magnification, 100 μm scale bars.

The integration of intrinsic and extrinsic apoptotic death signals depends on mitochondria. The major players in the process of apoptosis in mitochondria include the disruption of the mitochondrial membrane potential (ΔΨM) and the consequent release of cytochrome c from the mitochondria. The mechanism through which CAR induces apoptosis in HCT116 and HeLa cells was further assessed by analyzing the alterations in the ΔΨM within these cells. The decrease in the ΔΨM was evidenced by the decrease in JC-10 fluorescence in the mitochondria and the consequent increase in JC-10 green fluorescence in the cytosol due to the treatment with CAR (Figure 6A). A flow cytometry analysis indicated that in the CAR-treated HCT116 and HeLa cells after 48 h, the JC-10 green/JC-10 red ratio increased significantly; the most pronounced ΔΨM loss was observed in HeLa cells (4.1-fold) compared to HCT116 cells (1.78-fold) (Figure 6B). In order to better comprehend the role of CAR in inducing mitochondrial apoptosis, we employed immunofluorescence to examine the cytochrome c cellular distribution in cancer cells treated with CAR. Untreated cancer cells showed punctate patterns of cytochrome c, with some diffuse distribution in the cytoplasm, suggesting it was mainly present in the mitochondria. The release of cytochrome c from the mitochondria to the cytosol was significantly increased after CAR treatment of HCT116 and HeLa cells. The impact was demonstrated by the less punctate staining in the mitochondria and the enhanced diffuse staining in the cytoplasm. The effect on cytochrome c release was greater in HeLa cells (3.44-fold) than CAR-treated HCT116 cells (2.2-fold) and untreated cells (Figure 6C). These findings suggest that CAR may induce mitochondrial apoptosis by modifying ΔΨM and promoting the release of cytochrome c. This effect appears to be more significant in HeLa cells, eventually leading to cancer cell death.

### 3.7. CAR Induces Autophagy and Downregulates p62 in HCT116 and HeLa Cells

**Figure 7:**
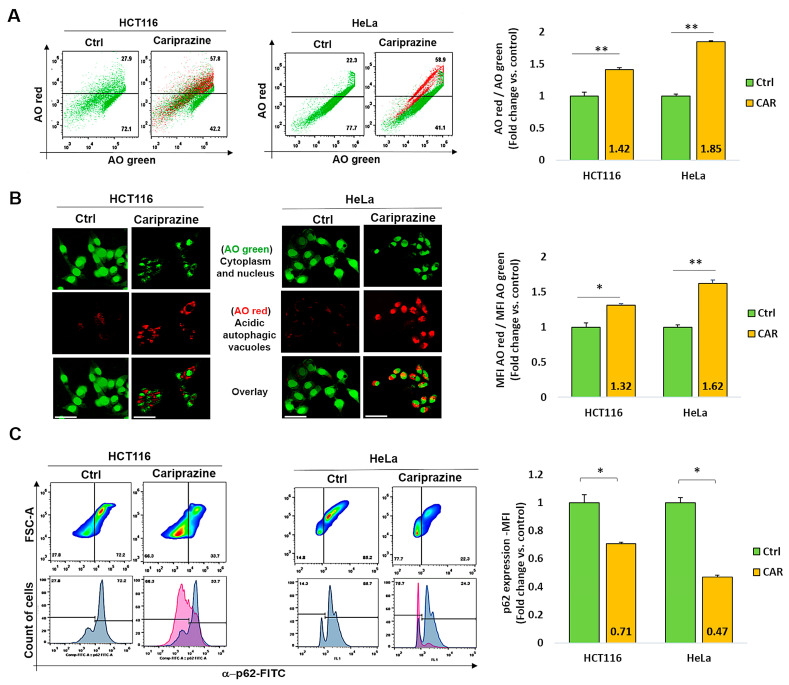
CAR represses p62 expression and triggers autophagy in HeLa and HCT116 cells. (**A**) Over the course of 48 h, cells were exposed to CAR (IC_50_—30 μM) before being stained with AO. The % of cells that generate AO-red or AO-green fluorescence was determined using flow cytometry. Bar graphs show mean % autophagy fold change ± SEM (*n* = 3). ** *p* < 0.01 in comparison to the control. (**B**) Red autophagic vacuoles can be seen in cancer cells that have been treated with CARs and stained with AO. AO red/AO green bar graphs show mean fold change in MFI ± SEM. Statistical significance: * *p* < 0.05 and ** *p* < 0.01 vs. control (*n* = 3). (**C**) CAR-treated cells were assessed for the protein expression (MFI) of the autophagic marker p62 using flow cytometry. The mean fold change of p62 MFI ± SEM (*n* = 3) was shown as histogram bars. * *p* < 0.05 in comparison to the control is statistically significant. Magnification 40×; 100 μm scale bars.

Numerous stress signals and medications, such as antipsychotics, have the ability to induce autophagy in different cancer cell types, which may result in apoptosis [45]. Thus, we examined the effect of CAR on autophagy regulation in HCT116 and HeLa cancer cells. Following a 48 h treatment with their corresponding CAR IC_50_ values (30 μM), the cancer cells were then labeled with acridine orange (AO). CAR’s treatment increased the percentage of autophagic cells to 57.8% in HCT116 cells and 58.9% in HeLa cells compared to the control (Figure 7A). Cells stained with AO verified the existence of autophagic vacuoles. Such an increase was indicated by a significantly higher level of AO red fluorescence in CAR-treated HeLa cells (1.62-fold) when compared to HCT116 cells (1.32-fold) and the control group (Figure 7B). Flow cytometry analysis also showed that the treatment with CAR reduced the levels of the autophagic marker p62 in HCT116 cells by 0.71-fold and in HeLa cells by 0.47-fold (Figure 7C). Hence, from these findings, it appears that CAR may have an important role in the induction of autophagy in cancer cells and that its activation in the case of HeLa cervical cancer cells is most pronounced.

### 3.8. CAR Inhibits Cell Cycle Progression in HCT116 and HeLa Cells

**Figure 8:**
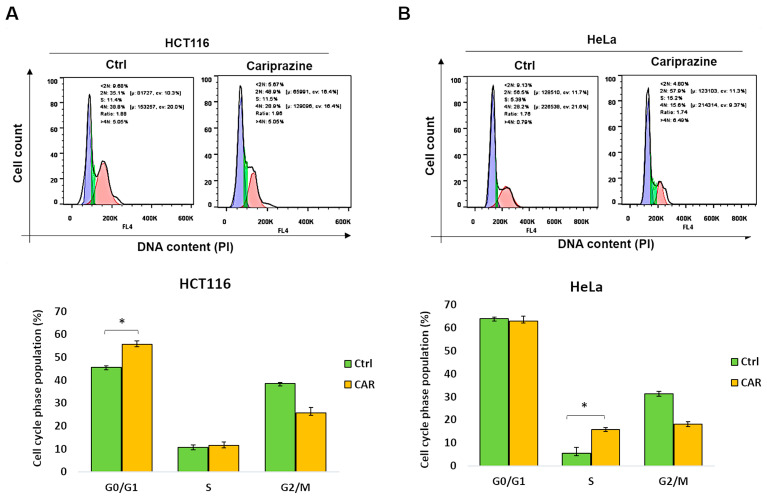
CAR causes HeLa and HCT116 cells to enter cell cycle arrest at various phases. (**A**) HCT116 and (**B**) HeLa cells, both untreated and treated with CAR (30 μM) for 48 h, were stained with propidium iodide (PI), and the phases of the cell cycle were analyzed by flow cytometry. The percentage of cells in the G0/G1, S, and G2/M phases was calculated by FlowJo software. The bars represent the mean % of cells in different phase ± SEM (*n* = 3). The results indicate that * *p* < 0.05 are statistically significant when compared to the control (ctrl).

Dysregulation of the cell cycle and DNA damage are common causes of tumor cell death. Therefore, we examined the distribution related to cell-cycle phases of CAR-treated HCT116 and HeLa cells following a period of 48 h, utilizing PI staining. The data showed that after CAR treatment, there was a significant rise (from 44.6% to 55.7%) in HCT116 cells in the G0/G1 phase (Figure 8A). In contrast, CAR-treated HeLa cells exhibited a substantial rise in the S cell cycle phase compared to the control (from 5.6% to 15.7%) (Figure 8B). These findings indicate that CAR can induce cell arrest in cancer cells, although the mechanism differs depending on the type of cancer.

### 3.9. CAR Decreases Phospho-Akt (Ser473) Expression in HCT116 and HeLa Cells

**Figure 9:**
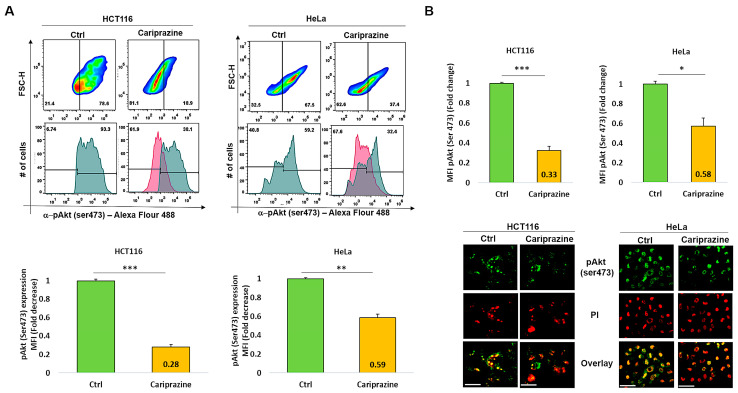
CAR suppresses p-Akt (Ser473) expression in HeLa and HCT116 cells. (**A**) The expression (MFI) of p-Akt (Ser473) was assessed by flow cytometry in both control and HeLa and HCT116 cells treated with CAR (IC_50_—30 μM) after 48 h. The bar graphs display the chosen HCT116 and HeLa cell populations that were employed to determine the MFI of p-Akt (Ser473). (**B**) The cellular location and intensity of green fluorescence (MFI) of p-Akt (Ser473) were evaluated using immunofluorescence (IF). The bars are plotted as the mean fold change in p-Akt MFI ± SEM (*n* = 3). * *p* < 0.05, ** *p* < 0.01 and *** *p* < 0.001 vs. control (scale bars = 100 µm; magnification 40×).

The PI3K/Akt signaling pathway may activate the cell survival mechanism, interfering with apoptosis. Therefore, we proceeded to evaluate the impact of CAR on p-Akt (Ser473) expression in HCT116 and HeLa cells, which were treated with their respective CAR IC_50_ values (30 μM) for 48 h. The exposure of the HCT116 cells to CAR resulted in a significant reduction in the expression level of p-Akt (Ser473) at 0.28-fold. The exposure of the HCT116 cells to CAR caused a significant reduction in the expression level of p-Akt (Ser473) to 0.28-fold. Despite a significant reduction in p-Akt, the HeLa cells exhibited a smaller decrease, showing a 0.59-fold reduction (Figure 9A). The immunofluorescence analysis aligned with these findings, indicating that the p-Akt’s mean fluorescence intensity (MFI) was considerably lowered in HCT116 cells treated with CAR, showing a 0.33-fold decrease, in contrast to the 0.58-fold reduction noted in HeLa cells (Figure 9B). Thus, these results indicate that CAR significantly reduced the levels of p-Akt (Ser473) in both HCT116 and HeLa cells; however, the effect was less pronounced in the cervical cancer cells.

### 3.10. In Silico PASS Prediction of CAR as a Potential Cytochrome bc1 Reductase Inhibitor

To better understand the mechanism underlying CAR-induced cell death in HeLa and HCT116 cells, we used the PassONLINE program to investigate its possible new biological activities. The results indicated a potential new biological activity of CAR as a ubiquinol-cytochrome bc1 reductase inhibitor, demonstrating a strong probability of being active (Pa) at 0.717. In contrast, the probability of being inactive (Pi) was recorded at 0.062, resulting in a Pa-Pi difference of 0.665 (Table 1). Notably, the predicted Pa value of CAR as a potential ubiquinol-cytochrome bc1 reductase inhibitor was higher than its Pa values associated with well-established biological activities as an antipsychotic and antidepressant drug, which were recorded at 0.596 and 0.507, respectively. Further MetaPASS analysis revealed that molecules structurally similar to CAR exhibit comparable predicted biological activity. This list includes well-known antipsychotics such as aripiprazole and domperidone, alongside diuron, a chemical herbicide recognized for its role as an inhibitor of cytochrome bc1 reductase, which is complex III of the mitochondrial respiratory chain (Supplementary Figure S1). It functions as a Q-cycle inhibitor that targets the ubiquinone (UQ) reduction site (Qi) of cytochrome bc1 reductase, disrupting the UQ—cytochrome bc1 complex [46,47,48]. Our results indicated that CAR was an effective inducer of mitochondrial apoptosis in the cancer cells we tested, especially in HeLa cells. Therefore, we selected the most promising new biological activity predicted for CAR from the PASS list, which is suggested as a potential cytochrome bc1 reductase inhibitor, for further molecular docking studies.

### 3.11. Retrieving the Structure of Cytochrome bc1 Reductase and Verifying the Qi Binding Site

**Figure 10:**
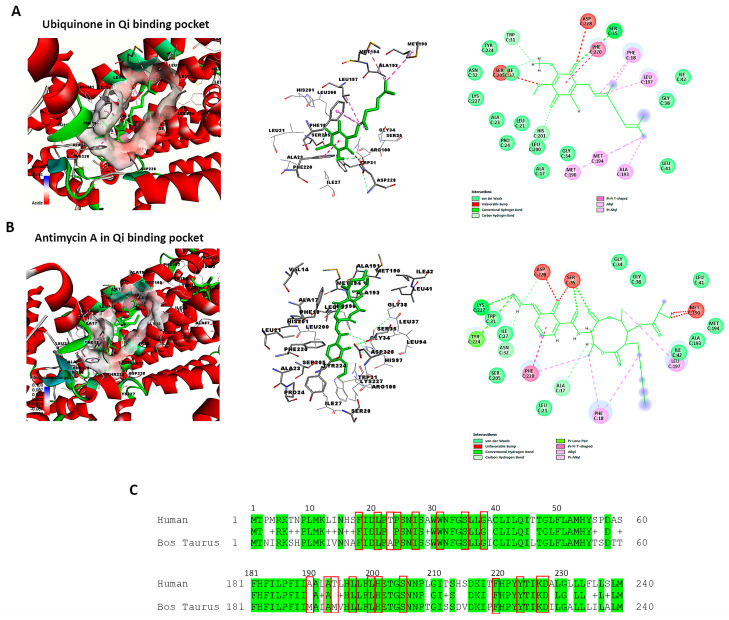
Identification of the Qi binding pocket of cytochrome bc1 reductase. This figure presents a detailed 3D view of the bovine Qi binding pocket of cytochrome bc1 reductase and 2D interactions between amino acid residues of Qi and (**A**) Ubiquinone (PDB:ID:1NTZ) and (**B**) Antimycin A (PDB: ID-1NTK). These interactions are elucidated using BIOVIA Discovery Studio Visualizer. (**C**) The sequence alignment compares bovine cytochrome b (UniProt P00157) to human cytochrome b (UniProt P00156). The identical residues between the two species are colored in green. The highly conserved interacting amino acid residues in the Qi binding pocket are highlighted in red boxes.

The active and binding site residues of Qi binding pocket in bovine cytochrome bc1 reductase have been identified and validated based on previous studies [30,49]. Using BIOVIA Discovery Studio Visualizer, we examined the crystal structure of cytochrome bc1 reductase in complex with UQ (PDB ID:1NTZ) at a resolution of 2.60 Å (Figure 10A), as well as the crystal structure of the mitochondrial cytochrome bc1 complex bound with Antimycin A1 (Ant A) (PDB ID: 1NTK) at the same resolution (Figure 10B). The Qi binding pocket of cytochrome bc1 reductase comprises essential amino acids that facilitate both the interaction with substrate UQ and the direct catalytic reduction in UQ, including Phe18, Leu21, Ala23, Pro24, Ile27, Trp31, Ser35, Gly38, Met190, Ala193, Met194, Leu197, Leu200, His201, Ser205, Phe220, Tyr224, Lys227, and Asp228 (Figure 10A). Furthermore, the amino acids at the Qi site that interact with the inhibitor Ant A, in addition to the previously mentioned residues, include Ala17, Asn32, Gly34, and Ile42 (Figure 10B). The examination of the sequence alignment between bovine cytochrome b (UniProt P00157) and human cytochrome b (UniProt P00156) showed 83% conservation at the Qi site, as highlighted in green in Figure 10C. Also, the interacting amino acid residues at the Qi site between humans and bovines have a high conservation rate of 90%, which is shown in red boxes in Figure 10C.

### 3.12. Molecular Docking of CAR to the Qi Site of Cytochrome bc1 Reductase

**Figure 11:**
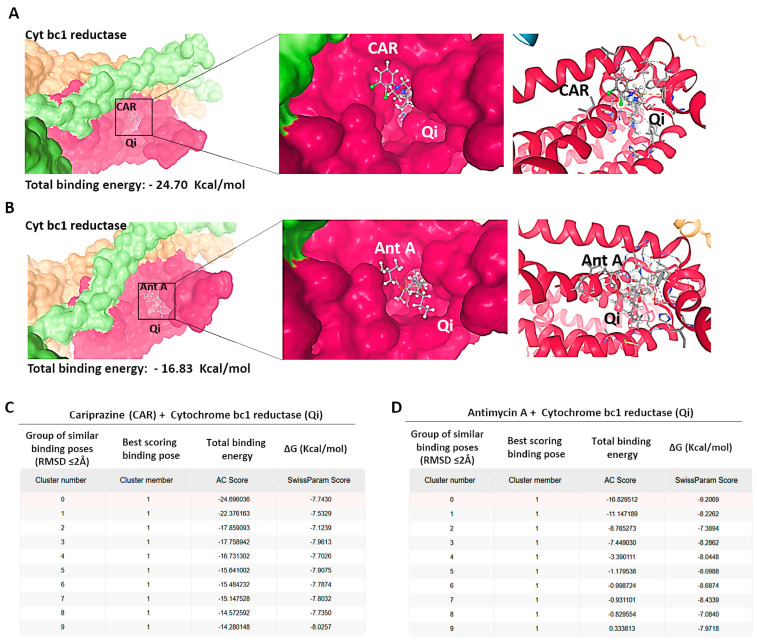
Binding predictions of CAR with the Qi binding pocket of cytochrome bc1 reductase. Molecular docking was conducted utilizing SwissDock 2024, and the structures of the most stable Qi binding site in cytochrome bc1 reductase complexes with (**A**) CAR and (**B**) Antimycin A (Ant A) are presented. The predicted total binding affinities are displayed as clusters of analogous poses, ranked according to the AC score (total binding energy), along with the estimated free energies (ΔG) for the binding interactions of (**C**) CAR with Qi site and (**D**) Ant A with Qi site.

To investigate the possible molecular interactions between CAR and the Qi site of cytochrome bc1 reductase (PDB ID: 1NTK), we employed molecular docking via the SwissDock 2024 server. This analysis predicted the possible CAR-Qi binding poses and estimated their binding affinities, allowing us to compare these results with the binding affinities of Ant A at the Qi site. The molecular docking results indicated that CAR interacts with the Qi site of cytochrome bc1 reductase (Figure 11A), similar to the interaction observed when Ant A is bound to the Qi site (Figure 11B). The predicted total binding affinities of CAR-Qi complex were presented as clusters of similar poses, ranked by AC score (total binding energy), ranging from −14.28 to −24.70 (Figure 11C). This result shows that CAR has a higher total binding energy than AntA-Qi, whose total binding affinities ranged from 0.33 to −16.83 (Figure 11D). The estimated free energies (ΔG) for all CAR-Qi clusters ranged from −7.13 to −8.19 Kcal/mol, which is comparable to the estimated free energies (ΔG) of AntA-Qi clusters, which ranged from −7.08 to −9.21 Kcal/mol (Figure 11C,D). These findings suggest that CAR may serve as a potential interacting ligand at the Qi site of cytochrome bc1 reductase.

### 3.13. CAR-Qi Complex Binding Analysis

**Figure 12:**
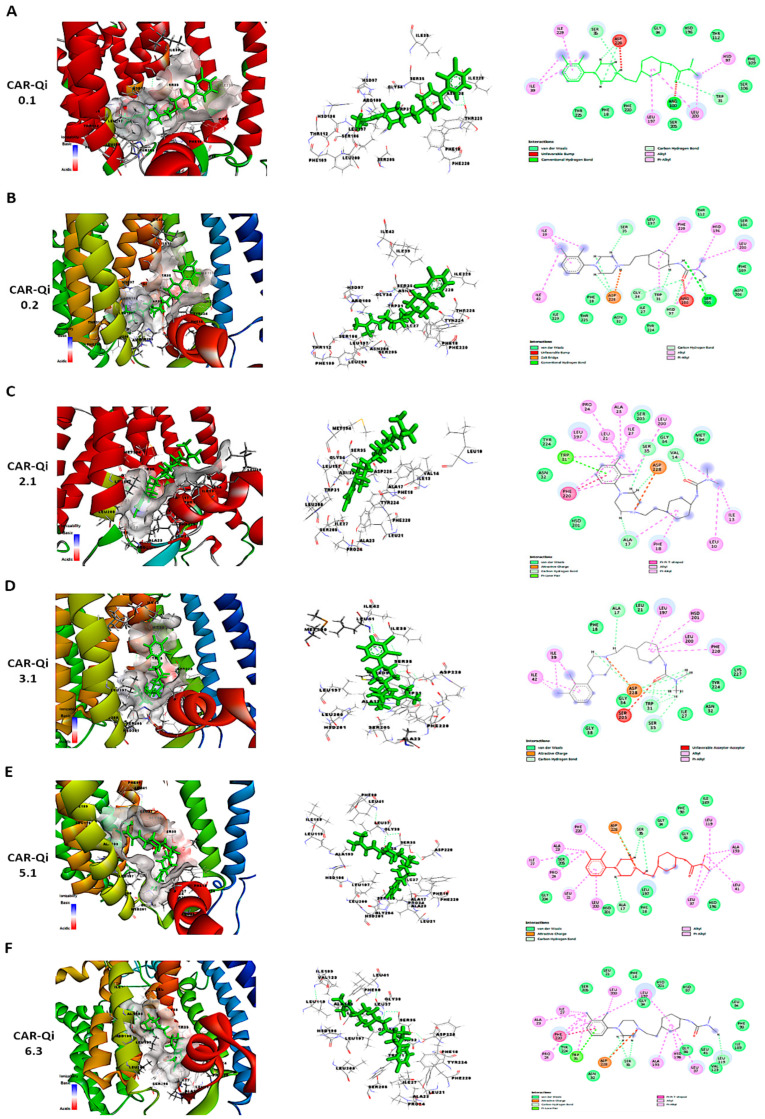
Three-dimensional and two-dimensional visualization of CAR’s interaction with cytochrome bc1 Qi site using BIOVIA Discovery Studio. The figure illustrates different binding poses and interactions of CAR-Qi clusters: (**A**) 0.1, (**B**) 0.2, (**C**) 2.1, (**D**) 3.1, (**E**) 5.1, and (**F**) 6.3. Predicted CAR interactions with Qi site amino acid residues; green dashed lines indicate hydrogen bonds, pink dashed lines represent hydrophobic pi–cation interactions, orange dashed lines denote attractive charge interactions, and light blue lines illustrate van der Waals interactions.

To further perform a post-dock analysis of CAR-Qi’s cytochrome bc1 reductase possible interactions, we utilized BIOVIA Discovery Studio 2025 to visualize and identify the significant chemical interactions between CAR and the Qi site, which are important for the confirmation of the stability and specificity of the CAR-Qi complexes. The most stable CAR–Qi complexes (CAR-0.1, 0.2, 2.1, 3.1, 5.1 and 6.3) were analyzed for their interactions based on their AC score (total binding energy) and the estimated free energy (ΔG in kcal/mol) derived from the docking results, including π-π stacking, hydrophobic contacts, hydrogen bonds, electrostatic bonds and van der Waals interactions. Additionally, we identified the important amino acid residues within the Qi site involved in these interactions (Figure 12). Each CAR-Qi complex analyzed demonstrated effective CAR binding within the Qi binding pocket. The CAR 6.3 complex exhibited the most favorable free binding energy at −8.19 Kcal/mol, followed by CAR 3.1 with −7.96 Kcal/mol, which was marginally lower than that of Ant A (−9.21 Kcal/mol), yet similar to that of UQ (−7.98 Kcal/mol). This suggests that CAR 6.3 has the strongest interaction with Qi compared to all the CAR complexes analyzed (Table 2).

It has been clearly established that UQ overlays the Qi site in cytochrome bc1 reductase, which is constituted by residues 17–39 and 193–229. The five most conserved residues involved in UQ binding are Asp228, His201, Gly34, Trp31, Ser35, and Lys227, while Leu197, Phe220, Ser205, Phe18, and Leu21 are significant for maintaining the stability of the binding [50]. All evaluated CAR complexes established interactions with particular amino acid residues within these residue domains. Each CAR complex created H-bonds with Asp228 and Ser35, whereas CAR 0.1, CAR 0.2, and CAR 3.1 also formed H-bonds with Trp31 (Figure 12 and Table 2), consistent with the H-bonds observed between UQ and the same amino acids. Furthermore, CAR 2.1, CAR 3.1, and CAR 5.1 formed H-bonds with Ala17, consistent with the H-bonds formed between inhibitor Ant A and the same amino acid (Figure 12C–E).

Additionally, CAR 2.1 and CAR 6.3 established strong π-π stacking interactions with Phe220, similar to those observed in UQ and Ant A binding. On the other hand, CAR 0.2, CAR 3.1, and CAR 5.1 showed π-alkyl interactions with Phe220. Furthermore, CAR 2.1, CAR 3.1, and CAR 6.3 formed hydrophobic alkyl interactions with Leu197, while CAR 2.1 also participated in π-alkyl interactions with Phe18, both of which were observed in UQ and Ant A binding. Likewise, CAR 5.1 and CAR 6.3 established interactions with Ala193 through alkyl hydrophobic bonds, similar to the interactions observed with Ant A (Figure 12 and Table 2).

Furthermore, although all CAR complexes demonstrated interaction with Gly34, only CAR 3.1 and CAR 6.3 exhibited interaction with Leu21 via van der Waals forces, analogous to UQ and Ant A (Figure 12D,F). Only CAR 0.2 established weak H-bonds with Gly34. Moreover, CAR 2.1, CAR 5.1, and CAR 6.3 engaged with Ser 205 via van der Waals interactions, similar to Ant A (Figure 12C,E,F), whereas only CAR 0.2 formed H-bonds with Ser 205, identical to UQ (Figure 12B). Although the binding of UQ to His 201 through H-bonds is crucial, neither of the CAR complexes formed H-bonds with His 201. However, only CAR 3.1 established hydrophobic π-alkyl interactions with His 201 (Figure 12D), while CAR 2.1, CAR 5.1, and CAR 6.3 interacted with His 201 through van der Waals forces (Figure 12C,E,F). It is important to mention that Ant A does not engage in any interactions with His 201. Interestingly, only CAR 3.1 interacted with Lys227 through van der Waals bonds, similar to UQ (Figure 12D), while Ant A forms strong H-bonds with Lys227. These results suggest that CAR may bind to the active region of the Qi binding site. The most efficient Qi site binding was by CAR 3.1 and CAR 6.3, which formed 19 favorable bonds with critical amino acid residues of Qi (14 and 16, respectively) (Table 2). This suggests that CAR may act as a potential inhibitor of mitochondrial cytochrome bc1 reductase.

### 3.14. Structural Comparison of CAR-Qi Complexes with Ant A-Qi and UQ-Qi Complexes

**Figure 13:**
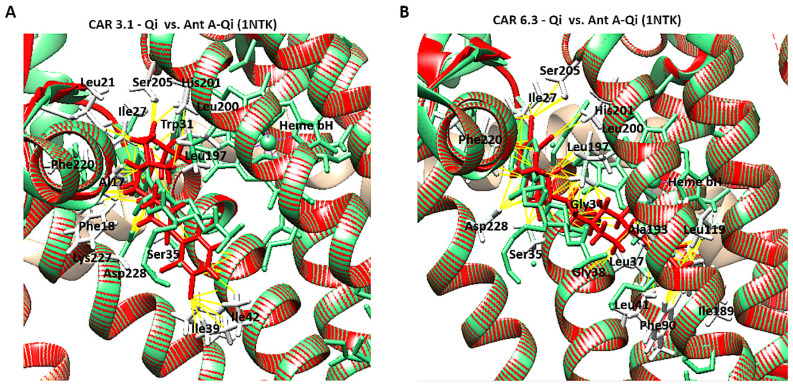
Structural comparison of CAR-Qi and Ant A-Qi complexes. Superimposition of the three-dimensional structures of (**A**) the CAR 3.1-Qi complex and (**B**) the CAR 6.3-Qi complex, obtained through molecular docking (red), with the crystal structure of the cytochrome bc1 Qi site bound to Antimycin A (Ant A) (PDB ID: 1NTK) (light green). Yellow dashes indicate the bonds formed between amino acids (white lines) and CAR at the QI site. Superimposition and visualization were performed utilizing UCSF Chimera.

To confirm accurate binding of CAR to the Qi active site, the CAR-Qi 3.1 and CAR-Qi 6.3 complexes, derived from molecular docking, were aligned with the crystal structure of Ant A-Qi cyt bc1 reductase complex (PDB ID: 1NTK). This structure was utilized as a template for homology modeling with UCSF Chimera. The analysis of the structures revealed that both CAR complexes showed superposition with the Ant A-Qi crystal structure. However, in the docked structure, the position and orientation of CAR 6.3 more closely resembled those of Ant A (Figure 13B) in the template crystal structure than those of the CAR 3.1 complex (Figure 13A). Moreover, the application of UCFS Chimera demonstrated the same interactions between CAR and the essential amino acids of the Qi binding site, which were similarly detected using BIOVA Discovery Studio.

**Figure 14:**
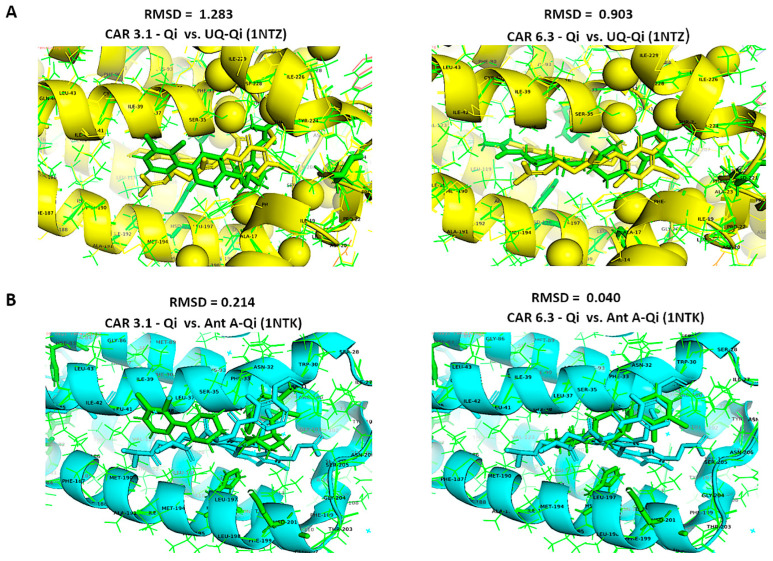
Structural alignments using the align function of PyMOL. Structural alignment and RMSD superimposition of predicted CAR-Qi complexes (green) with (**A**) the native UQ-Qi complex (PDB ID: 1NTZ) (yellow) and (**B**) the native Ant A-Qi complex (PDB ID: 1NTK) (blue).

Furthermore, we aligned the crystal structures of the native UQ-Qi cyt bc1 reductase (PDB ID: 1NTZ) and Ant A–Qi cyt bc1 reductase (PDB ID: 1NTK) with the predicted models CAR-Qi 3.1 and CAR-Qi 6.3 using PyMOL software. This alignment aimed to assess the structural similarity between the native and predicted ligand–enzyme complex structures. When comparing the native UQ-Qi complex to the predicted structures, the results indicated RMSD values of 0.903 for the CAR 6.3 complex and 1.283 for the CAR 3.1 complex (Figure 14A). The alignment of the Ant-Qi complex with CAR 6.3 and CAR 3.1 yielded RMSD values of 0.040 and 0.214, respectively (Figure 14B). These results suggest a high degree of similarity between the native and predicted structures. A slightly greater similarity was noted between the CAR 6.3-Qi and UQ-Qi and Ant-A-Qi complexes, confirming the accurate prediction of CAR 6.3’s binding to the Qi binding site. Overall, our findings support the hypothesis that CAR may interact with the Qi site of complex III.

### 3.15. CAR Induces ROS Production

**Figure 15:**
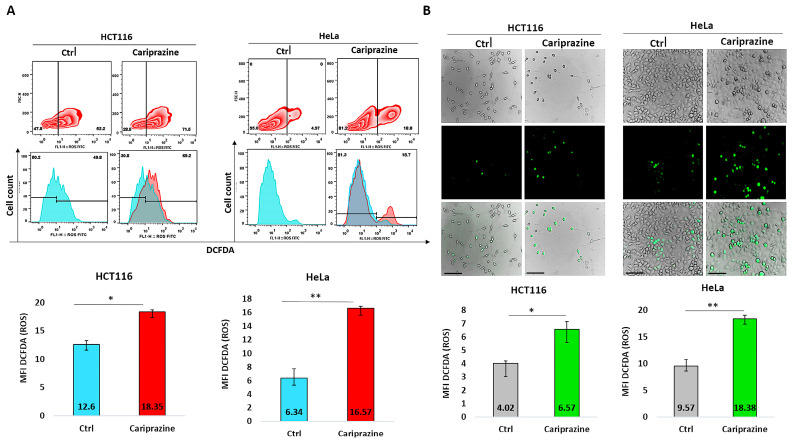
Quantification of reactive oxygen species (ROS) production using the DCFDA assay. (**A**) Flow cytometry was used to analyze ROS production via DCFDA in untreated and CAR (IC_50_—30 μM)-treated HCT116 and HeLa cells after 48 h. The histograms represent the selected population of HCT116 and HeLa cells used to determine the MFI of DCFDA (green fluorescent). (**B**) The green fluorescence intensity (MFI) of DCFDA was measured using immunofluorescence (IF) in HCT116 and HeLa cells treated with CAR (IC_50_). The bars show the MFI of DCFDA ± SEM (*n* = 3). Levels of significance: * *p* < 0.05 and ** *p* < 0.01 compared to the control. At a magnification of 40×, the scale bars measure 100 μm.

To further support our findings obtained from CAR’s induction of mitochondrial apoptosis and the molecular docking studies suggesting that CAR may act as a potential inhibitor of the Qi-site of cytochrome bc1 reductase, we conducted the DCFDA assay to examine the effect of CAR on ROS production in HCT116 and HeLa cells. The results from the DCFDA assays indicated that CAR induced ROS generation in both cell lines. However, the effect on ROS production was significantly greater in HeLa cells, which showed an increase of approximately 2.63-fold compared to HCT116 cells, which increased by around 1.46-fold, as determined by flow cytometry (Figure 15A). Image fluorescence analysis demonstrated similar results, showing a 1.92-fold increase in ROS for HeLa cells compared to the control and a 1.62-fold increase for HCT116 cells. Moreover, CAR-treated HCT116 cells showed reduced ROS production in comparison to HeLa cells, yet they displayed more significant morphological apoptotic changes, including rounding and shrinkage, indicating a higher percentage of dead cells relative to CAR-treated HeLa cells (Figure 15B).

## 4. Discussion

This study is noteworthy since it is one of the first to assess the intricate cellular mechanisms and effects associated with CAR-induced cell death in the context of cancer treatment involving HCT116 colon and HeLa cervical cells, and it suggests that CAR may act a potential Qi-site inhibitor of cytochrome bc1 reductase (complex III). The choice of CAR for mechanistic investigation of its cytotoxic effects as a potential Qi-site inhibitor of cytochrome bc1 reductase was guided by our prior research demonstrating its anticancer activity in TNBC cells via activation of mitochondrial apoptosis [16], existing evidence indicating its capacity, as well as that of other antipsychotics, to inhibit the ETC [31], and our preliminary PASS predictions suggesting that Qi-site cytochrome bc1 reductase inhibition may suggest its potential mechanism of action (Table 1).

**Figure 16:**
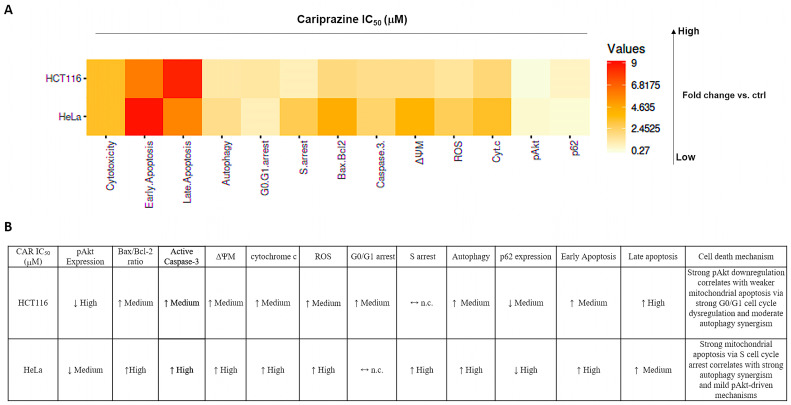
Cariprazine influences multiple cell signaling pathways, such as apoptosis, autophagy, and cell cycle regulation, with different levels of intensity in HeLa and HCT116 cells. (**A**) The heatmap displays the relative fold changes in apoptosis, autophagy, cell cycle arrest, and critical associated protein expressions in HCT116 and HeLa cells after 48 h of treatment with CAR (IC_50_—30 μM) compared to the control group. (**B**) A table illustrating the relationships between apoptosis, autophagy, cell cycle arrest, and their primary protein markers following CAR treatment of HCT116 and HeLa cells. ↓ represents high fold change; ↑ represents medium fold change; ↔ n.c. represents no change compared to control.

Our findings showed that CAR promoted apoptosis in both HeLa and HCT116 cancer cells, but there were significant variations in mitochondrial dysfunction, apoptotic signaling, autophagy induction, and cell cycle arrest (Figure 16). These differences may be mechanistically associated with CAR’s predicted potential to inhibit the Qi site of mitochondrial cytochrome bc1 reductase, as proposed by PASS analysis, docking binding energies, and structural alignment with ubiquinone (UQ) and antimycin A (Ant A), a recognized Qi-site inhibitor (Figure 11, Figure 12, Figure 13 and Figure 14; Table 2). Together, our results collectively suggested that CAR may act as an inhibitor at the Qi-site of mitochondrial complex III, causing increased mitochondrial dysfunction and apoptosis, particularly in HeLa cells. Importantly, the hypothesis of CAR’s inhibition of the Qi site is predictive and relies on the convergence of in silico predictions with complex III inhibition-specific mitochondrial characteristics (ROS production, cytochrome c release, and ΔΨm loss).

First, CAR exhibited antitumor effects in HeLa and HCT116 cancer cells primarily by stimulating apoptosis. Through our integrative research, we were able to determine that CAR’s apoptotic activity is supported by an interconnected network of cellular responses in these two distinct cancer cells (Figure 16). CAR’s treatment led to a significant increase in the population of apoptotic cells. Specifically, there was a significantly higher occurrence of early apoptosis in HeLa cells compared to HCT116 cells, while HCT116 cells exhibited greater late apoptosis than HeLa cells. Moreover, CAR induced significantly higher mitochondrial membrane depolarization (ΔΨm loss), a more substantial increase in cytosolic cytochrome c and ROS production in HeLa cells than in HCT116 cells. The differences in CAR apoptotic patterns indicate that apoptosis in HeLa cells occurs rapidly as a result of acute mitochondrial damage. Conversely, CAR triggered apoptosis in HCT116 cells at a slower rate, potentially as a result of partial mitochondrial inhibition or the activation of alternative metabolic pathways in colorectal carcinoma cells. This cell-specific difference aligns well with our hypothesis that CAR may potentially inhibit the Qi site of cytochrome bc1 reductase. Other researchers also demonstrated that antipsychotics, such as zotepine, aripiprazole, quetiapine, and risperidone, could significantly inhibit cytochrome bc1 reductase (complex III), which could initiate the mitochondrial apoptotic pathway [30]. Classic Qi-site inhibitors like antimycin A exert significant mitochondrial apoptosis in cell types that heavily rely on complex III-coupled oxidative phosphorylation.

This ultimately results in considerable ROS generation, a collapse of ΔΨm, and an increased release of cytochrome c [51,52,53]. Our results showed that the binding affinities of CAR to the Qi site of complex III produced docking binding energies that were close to those of Ant A (ΔG = −7.96 to −8.19 kcal/mol, compared to −9.21 kcal/mol for Ant A). Additionally, the structural superimposition of CAR and Ant A within the Qi pocket of cytochrome bc1 reductase provides support to these findings (Figure 11, Figure 12, Figure 13 and Figure 14). Furthermore, our findings correspond with previous research revealing the inhibition of the mitochondrial Qi-site by Ant A, wherein early apoptotic signaling was coupled with ΔΨm disruption and cytochrome c release, which were more pronounced in HeLa cells [54]. In line with this, previous research indicated that the expression of mitochondrial cytochrome bc1 reductase was lower in colon tumor tissues compared to normal tissues and was further reduced in metastatic tumors compared to primary tumors [55]. This reduction may enable HCT116 cells to metabolically shift, adapt, and survive under stress, including during the early stages of apoptosis. These data collectively suggest that cervical tumor cells are more dependent on oxidative phosphorylation and thus would be more vulnerable to CAR’s activation of mitochondrial apoptosis than colon HCT116 cancer cells, which exhibit a classical Warburg phenotype [56].

Furthermore, our findings demonstrated that CAR induced a more substantial increase in the Bax/Bcl-2 ratio and elevated levels of caspase-3 activation in HeLa cells relative to HCT116. These greater CAR apoptotic signals in HeLa cells are associated with a greater extent of mitochondrial damage, which is consistent with expectations for a Qi-site inhibitor. Antimycin A-like inhibitors are recognized for their ability to enhance ROS production by blocking mitochondrial electron transport at the Qi site of cytochrome bc1 reductase. This inhibition enhances Bax activation, causes the permeabilization of the mitochondrial outer membrane, and ultimately results in the activation of caspase 3. Earlier research showed that Ant A efficiently induced apoptosis in juxtaglomerular cell tumor cells (As4.1 cells), which was associated with a loss of ΔΨm, a decrease in Bcl-2 levels, activation of caspase-3, and cleavage of PARP [57]. Interestingly, Ant A has also been identified as an effective inhibitor of Bcl-2, and various analogues of Ant A have demonstrated a strong ability to bind to the antiapoptotic protein Bcl-2. These analogues inhibit the antiapoptotic function of Bcl-2 in cancer cells that overexpress this protein, such as those found in breast cancer and HeLa cells [58].

Furthermore, a more recent study demonstrated that the novel Ant A analog, Antimycin A2c, effectively inhibited complex III of the electron transport chain in HeLa cells. This inhibition caused mitochondrial dysfunction, an increase in Bax/BAK levels, a decrease in Bcl-2 expression, and the activation of caspase 9 and caspase 3, ultimately resulting in apoptosis [59]. Our results align with previous research indicating that HeLa cells exhibit a greater dependence on oxidative phosphorylation for survival compared to HCT116 cells, perhaps enhancing the apoptotic effect of CAR. In HCT116 cells, which primarily depend on glycolytic metabolism for survival, the combination of antimycin A and the glucose metabolism inhibitor, 2-deoxy-D-glucose (2DG), significantly increased the activation of mitochondrial apoptosis [60]. In fact, treating HCT116 cells with Ant A for 7 days induced apoptosis, increased caspase 3 activation, and upregulated the mRNA levels of caspase 9 and p53 [61]. Furthermore, the results of our docking study indicated that CAR was capable of forming H-bonds with important Qi-site amino acids, namely Asp228, Ser35, Trp31, and Ser205. Our docking data also demonstrated that CAR formed hydrophobic stacking interactions with Phe220, Leu197, and Phe18, along with other interactions analogous to those identified with UQ and Ant A bound to the Qi-site (Figure 12, Figure 13 and Figure 14; Table 2). These findings provide additional evidence for the hypothesis that CAR may interfere with Qi-mediated electron flow during oxidative phosphorylation, leading to the activation of Bax and caspase 3, which, in turn, promotes mitochondrial apoptosis, especially in HeLa cells.

Furthermore, CAR significantly increased autophagy in both cell types, but it contributes differentially to cell death in both cell lines. Namely, the overall autophagic flux induced by CAR, as indicated by AO red fluorescence and p62 degradation, was more pronounced in HeLa cells compared to HCT116 cells (p62 was reduced to 0.47-fold in HeLa vs. 0.71-fold in HCT116). Autophagy is a well-established secondary response to mitochondrial stress-induced apoptosis [62,63]. Different antipsychotic medications, including atypical drugs like aripiprazole, clozapine, olanzapine, quetiapine, and risperidone, have demonstrated anticancer effects through various mechanisms of cell death, such as autophagy, in several types of cancer cells, including those associated with cervical and colon cancers [64]. Furthermore, cancer cells may be able to remove damaged mitochondria and activate autophagy if ROS accumulation and impaired electron transport are the driving forces behind inhibition of oxidative phosphorylation, particularly complex I and complex III of the mitochondria [65,66]. Aside from its effects on cancer cells, Ant A has been demonstrated to induce autophagy in a human retinal pigment epithelial (RPE) cell line (ARPE-19 cells) while also activating mitochondrial apoptosis. This suggests that the increased production of ROS by Ant A may play a significant role in regulating autophagy [67]. The effect of Ant A on inducing autophagy was further demonstrated by using autophagy mutant cells with deletions in specific genes that code for autophagy-related proteins, which showed a complete block of Ant A-induced autophagy [68]. Given CAR’s predicted and potential docking modeled binding at the Qi site, similar to Ant A, in HeLa cells, CAR-induced autophagy appears to act as a pro-death mechanism. This observation corresponds with the considerable reduction in ΔΨm and the increased generation of ROS observed in HeLa cells, both of which facilitate the process of apoptosis. In HCT116 cells, CAR-activated autophagy appears to function as an adaptive response that delays apoptosis until there is an accumulation of mitochondrial damage.

Furthermore, CAR induced distinct patterns of cell cycle arrest in HeLa and HCT116 cells, indicating that the mechanisms driving mitochondrial stress-induced apoptosis vary between these two cell types. CAR significantly caused G0/G1 phase arrest in HCT116 cells (44.6% in control compared to 55.7% in CAR-treated cells), while it primarily induced S phase arrest in HeLa cells (5.6% in control versus 15.7% in CAR-treated cells). These distinct cell cycle arrest patterns signify essential variations in the response of each cell type to mitochondrial damage, ROS signaling, and ATP depletion, which are characteristic of Qi-site inhibition of complex III. In HeLa cells, CAR’s induction of S phase arrest was associated with significant mitochondrial injury and a rapid early intrinsic apoptosis. This response is typical of strong Qi-site inhibition and is similar to the phenotypes that antimycin A causes. Our findings indeed correspond with earlier research indicating that Ant A caused mitochondrial damage, resulting in oxidative stress that interfered with cell cycle machinery and ultimately arrested HeLa cells in the S-phase, thereby inhibiting further proliferation [69]. It has been well established that the S cell cycle phase is highly ATP-dependent [70,71]. Therefore, the S-phase arrest triggered by CAR in HeLa cells was linked to a rapid initiation of mitochondrial apoptosis. The observed effect may be associated with the predicted Qi-dependent dysfunction of the mitochondrial ETC induced by CAR, leading to increased ROS production and a possible decrease in ATP generation. On the other hand, CAR induced G0/G1 phase arrest in HCT116 cells, which was linked to mild mitochondrial dysfunction, decreased ROS generation, and delayed apoptosis. These data indicate that in CAR-treated colon cancer cells, cell cycle arrest may occur before mitochondrial apoptosis, unlike in HeLa cells, where apoptosis occurs immediately. Ant A has been shown to arrest cancer cells in the G0/G1 phase, including the human lung cancer cell line Calu-6. This arrest subsequently resulted in the activation of mitochondrial apoptosis and an elevation in Bax and caspase-3 activation. Moreover, the authors observed that while Ant A successfully induced mitochondrial apoptosis, the total production of ROS remained minimal, consistent with our results obtained in CAR-treated HCT116 cells [72]. Our findings are supported by research indicating that the antipsychotic drug trifluoperazine (TFP) induces G0/G1 cell cycle arrest, subsequently activating mitochondrial apoptosis in human colon cancer cells SW620 and HCT116, as well as in the mouse colon cancer cell line CT26 [73]. A recent study has demonstrated that the antipsychotic clozapine can induce G0/G1 cell cycle arrest and increase ROS production, which is accompanied by apoptosis and autophagy in MCF-7 and MDA-MB-231 breast cancer cells [6].

Furthermore, although CAR reduced Akt phosphorylation in both cell lines, the decrease was larger in HCT116 (0.28-fold) than in HeLa (0.59-fold) cells. This suggests that CAR’s mitochondrial cell death mechanism interacts differently with the Akt survival pathway in these cancer cells. For HCT116, which revealed less mitochondrial damage, CAR might inhibit survival signaling more strongly to compensate for a lower mitochondrial pro-apoptotic drive and late apoptosis. Conversely, in HeLa, survival signaling is reduced but not as strongly as in HCT116; so, mitochondrial collapse is the dominant cell death mechanism, which is coupled with strong early apoptosis. Another antipsychotic drug, thioridazine, has also been shown to induce both early and late apoptosis through regulation of the PI3K/Akt/mTOR pathway in cervical (HeLa, C33A, and Caski) and endometrial (HEC-1-A and KLE) cancer cells [74]. In accordance with CAR’s in silico study predicting a potential role as a Qi-site cytochrome bc1 reductase inhibitor, additional studies demonstrated that an Ant A analog, an alkaloid known as NADA, induced mitochondrial dysfunction and ROS-mediated apoptosis. This was accompanied by a significant increase in the Bax/Bcl-2 ratio, activation of caspase 3, and reduced phosphorylated levels of PI3K, Akt, and mTOR in HeLa cells [75]. Furthermore, another study demonstrated that the PKCδ-selective activator (Roy-Bz) reduced mitochondrial respiration in complexes I and III in colon HCT116 cancer cells, as well as glycolysis, effectively inducing apoptosis in these cells. This finding aligns with our results, suggesting that the survival of HCT116 cells strongly relies on enhanced metabolic resilience and survival signaling pathways, which delay the activation of apoptosis by CAR [76].

It appears that the cytotoxic and apoptotic effects exerted by the CAR in this study could be attributed to its molecular structure and its similarity to other antipsychotics that demonstrate anticancer activity. Cariprazine has structural similarities with other antipsychotics such as aripiprazole and brexpiprazole in terms of having a lipophilic heterocyclic moiety, presence of aromatic rings, and protonatable nitrogen [77]. These properties are known to promote mitochondrial accumulation driven by the mitochondrial membrane potential, to favor interactions with hydrophobic protein pockets within the inner mitochondrial membrane [78], and to subsequently affect mitochondrial function by inducing mitochondrial depolarization, ROS generation, and oxidative stress, as demonstrated with aripiprazole [79].

Importantly, several antipsychotics with a similar structure to that of CAR have been shown to impair mitochondrial respiration, increase ROS production, and activate intrinsic apoptosis and/or autophagy pathways in cancer cells. All of these effects are consistent with mitochondrial electron transport chain complex interference, including complex III [30]. In line with our results, a previous study showed that aripiprazole could efficiently activate autophagic apoptosis in colorectal cancer [80], as well as mitochondrial apoptosis in pancreatic cancer cells [13]. Furthermore, brexpiprazole has been demonstrated to exert an efficient antitumor effect in cancer cells and cancer stem cells in glioblastoma, pancreatic cancer, and lung cancer through activation of mitochondrial apoptosis [81]. Moreover, aripiprazole, brexpiprazole and cariprazine were demonstrated to efficiently inhibit mitochondrial respiratory complex I [32]. Thus, the results obtained in our study are in accordance with the previously published data regarding the antipsychotic structurally and pharmacologically similar to cariprazine. In our study, cariprazine induced key features of mitochondrial apoptosis, such as loss of mitochondrial membrane potential, cytochrome c release, ROS generation, Bax/Bcl-2 imbalance, and caspase-3 activation, pathways that are well documented as characteristics associated with the inhibition of mitochondrial electron transport chain (ETC) function.

## 5. Conclusions

Together, our results suggest a molecular hypothesis in which CAR may bind to cytochrome bc1 reductase’s Qi site, disrupting electron transport, increasing mitochondrial stress, and triggering apoptosis. The mitochondrial apoptotic cascade is more evident in HeLa cells, associated with a more favorable structural alignment of CAR with Ant A and UQ at the Qi binding pocket, along with a more significant collapse of mitochondrial function. At the same time, CAR-treated HCT116 cells undergo apoptosis due to a combination of moderate mitochondrial inhibition, G0/G1-phase arrest, and significant p-Akt suppression. This study therefore suggests the first supportive in silico evidence that CAR may act as a novel antitumor agent as a potential Qi-site inhibitor that disrupts complex III function and drives a mitochondria-dependent apoptosis, with cell-type-specific differences in metabolic reliance and downstream signaling intensity. In the future, confirmation of the interaction between CAR and the Qi site through direct enzymology and bioenergetic rescue experiments is essential. Moreover, the compelling demonstration of Qi-site inhibition by CAR will require additional functional studies in the future, such as direct measurements of complex III enzymatic activity, analysis of oxygen consumption rate, or rescue tests employing alternate electron donors or known Qi-site inhibitors. These future findings, along with our results from this investigation, might provide support to the idea that Qi inhibition may be the molecular mechanism driving CAR’s potential as a mitochondrially targeted anticancer agent.

## Figures and Tables

**Table 1:** Prediction of biological activities of CAR by PASS software.

Pa	Pi	Cariprazine—Biological Activity
0.884	0.005	Antineurotic
0.681	0.005	5 Hydroxytryptamine antagonist
0.730	0.063	Phobic disorders treatment
0.717	**0.062**	**Ubiquinol-cytochrome-c reductase inhibitor**
0.596	0.012	Antipsychotic
0.508	0.020	Antidepressant
0.507	0.021	Mood disorders treatment
0.489	0.027	Sigma receptor agonist
0.437	0.013	Antialcoholic
0.467	0.056	Fibrinogen receptor antagonist
0.397	0.009	HERG channel blocker
0.398	0.012	Dependence treatment
0.505	0.120	CYP2H substrate
0.381	0.011	Alpha adrenoreceptor antagonist
0.488	0.141	Nootropic
0.352	0.009	Alpha 1a adrenoreceptor antagonist
0.401	0.059	CYP2D6 substrate
0.377	0.037	Neuropeptide Y2 antagonist
0.361	0.024	Proto-oncogene tyrosine-protein kinase Fgr inhibitor
0.369	0.037	Antiparkinsonian
0.435	0.106	Gastrin inhibitor
0.334	0.011	Alpha 1 adrenoreceptor antagonist
0.333	0.014	Raynaud’s phenomenon treatment
0.382	0.064	CYP2D substrate
0.327	0.011	5 Hydroxytryptamine 1 antagonist
0.367	0.055	HERG 1 channel blocker
0.408	0.103	Antinociceptive
0.311	0.011	Cocaine dependency treatment
0.304	0.005	Neuropeptide Y antagonist
0.302	0.004	Dopamine D2S antagonist
0.310	0.015	Antiadrenergic
0.309	0.016	5 Hydroxytryptamine 2 antagonist
0.301	0.010	5 Hydroxytryptamine 1A antagonist
0.303	0.015	Adrenaline antagonist
0.329	0.043	Antiparkinsonian, rigidity relieving
0.394	0.118	Neurotransmitter uptake inhibitor
0.303	0.042	5 Hydroxytryptamine 1E antagonist
0.318	0.062	Anxiolytic
0.358	0.144	Phospholipid-translocating ATPase inhibitor
0.328	0.131	CYP3A4 substrate
0.377	0.185	Mucomembranous protector
0.381	0.201	Anti-ischemic, cerebral
0.328	0.162	Anaphylatoxin receptor antagonist
0.309	0.143	Spasmolytic, urinary
0.316	0.163	APOA1 expression enhancer
0.311	0.196	Heat shock protein 27 antagonist

**Table 2:** Docking and ligand binding affinities, binding sites, the number and types of bonds between amino acids at the Qi site with various CAR clusters, Antimycin A, and Ubiquinone. In the Qi site, amino acids in bold interact with CAR and Ubiquinone, while underlined amino acids bind CAR and Ant A.

Interaction with Qi Binding Site	Total Binding Affinity (AC Score)	Estimated Free Binding Energy (ΔG) Kcal/mol	Amino Acids Residues Forming H-Bonds	Amino Acids Residues Forming Hydrophobic Bonds	Amino Acids Residues Forming Electrostatic Bonds	Amino Acids Residues Forming van der Waals Interactions
CAR 0.1 cluster	−24.7	−7.74	5—Arg100, **Asp228**, **Ser35**, **Trp31**	7—Leu200, Ile39, Leu197, Ile229, Hsd97		9—**Gly34**, Hsd196, Thr112, Phe109, Ser106, Ser205, Phe220, Phe18, Thr225
CAR 0.2 cluster	−20.59	−7.79	8—**Ser205**, **Trp31**, **Asp228**, **Ser35**, Gly34, Hsd97	9—Leu200, Ile39, Ile42, Trp31, Hsd97, Hsd196, **Phe220**	1—Asp228	11—Leu197, Thr112, Ser106, Phe109, Asn206, **Ile27**, **Tyr224**, **Asn32**, Thr225, Phe18, Ile229
CAR 2.1 cluster	−17.86	−7.13	4—**Asp228**, **Ser35**, Ala17, Val14	17—Trp31, **Phe220**, Leu10, Ile13, Val14, Ala23, Pro24, Ile27, Leu21, **Leu197**, Leu200, Ala17, **Phe18**	1—Asp228	6—**Tyr224**, **Asn32**, Hsd201, **Ser205**, **Gly34**, Met194
CAR 3.1 cluster	−17.76	−7.96	10—**Asp228**, **Trp31**, Ala17, **Ser35**	9—Ile39, Ile42, **Leu197**, Leu200, Hsd201, **Phe220**	1—Asp228	8—Phe18, **Leu 21**, **Tyr224**, **Lys 227**, **Asn32**, **Ile27**, **Gly34**, **Gly38**
CAR 5.1 cluster	−15.64	−7.91	3—Ala17, **Asp228**, **Ser35**)	12—Leu37, Leu41, **Ala193**, Leu119, Ala23, Pro24, Leu21, Leu200, **Phe220**, Ile27	1—Asp228	10—Ser205, Gly204, Hsd201, Phe 18, Leu197, Hsd196, Ile189, **Gly38**, Phe90, **Gly34**
CAR 6.3 cluster	−13.14	−8.19	4—**Asp228**, **Ser35**, Leu119	15—Trp31, **Phe220**, Ala23, Pro24, Ile27, **Leu197**, Leu200, Leu37, **Ala193**, Hsd196	1—Asp228	14—Ser 205, **Leu21**, Phe18, **Gly34**, Hsd201, Hsd97, Leu94, Phe90, Ile189, Val123, **Leu 41**, **Gly38**, **Asn 32**, **Tyr 224**
Antimycin A	−16.83	−9.21	11—Lys227, Asn32, Asp228, Ser35, Ala17, Gly38, Met194	6—Tyr224, Phe220, Phe18, Leu197		10—Trp31, Ile27, Ser205, Leu21, Ile42, Ala193, Leu41, Gly34
Ubiquinone	−25.1	−7.98	6—Ser35, Trp31, Ser205, His201 (Asp228)	7—Phe220, Ala193, Leu197, Met190, Met194, Phe18		13—Tyr224, Asn32, Lys227, Ala23, Leu21, Pro24, Leu200, Ala17, Gly34, Leu41, Gly38, Ile42, Ile27

## Data Availability

The original contributions presented in this study are included in the article/Supplementary Material. Further inquiries can be directed to the corresponding authors.

## Supplementary Materials

The following supporting information can be downloaded at: https://www.mdpi.com/article/10.3390/biomedicines14020315/s1, Figure S1: MetaPASS application (https://way2drug.com/MetaPASS/, accessed on 16 October 2025) was used for analyzing the biological activity spectrum of organic compounds using CAR as an original ligand to find similar structure compounds with similar or same biological activity, taking into account their biotransformation (metabolic pathways).

## Abbreviations

The following abbreviations are used in this manuscript:

CAR	Cariprazine
PI3K	Phosphoinositide 3-kinase
AMPK	AMP-activated protein kinase
ROS	Reactive oxygen species
ABCG2	ATP-Binding Cassette Subfamily G Member 2
TNBC	Triple-negative breast cancer
ETC	Electron transport chain
ΔΨm	Mitochondrial membrane potential
ATP	Adenosine three phosphate
DMEM	Dulbecco’s Modified Eagle’s Medium
FSB	Fetal bovine serum
5-FU	5-fluorouracil
AO/EtBr	Acridine orange/ethidium bromide
PBS	Phosphate-buffered saline
DCFDA	Dichloro fluorescein diacetate assay
MFI	Mean fluorescence intensity
PASS	Predicted Activity Spectrum for Substances
7-AAD	7-Aminoactinomycin D
PI	Propidium iodide
UQ	Ubiquinone
Ant A	Antimycin A
ΔG	Gibbs free energy change
