# Supplementary material for: Antipsychotic Drug Cariprazine Induces Distinct Cell Death Mechanisms in HeLa and HCT116 Cells as a Potential Inhibitor of Qi-Site of Cytochrome bc1 Reductase
Source: Biomedicines. 2026 Jan 30;14(2):315. doi: 10.3390/biomedicines14020315 (PMC12938579; doi:10.3390/biomedicines14020315)
Supplement: Supplementary file 1 [file biomedicines-14-00315-s001.zip › biomedicines-4092714-supplementary.pdf]

Supplementary Figure S1. MetaPASS application (https://way2drug.com/MetaPASS/) was used for analyzing the biological activity spectrum of organic compounds using CAR as an original ligand to find similar structure compounds with similar or same biological activity, taking into account their biotransformation (metabolic pathways).

**Compound**	**Name**	S_{MNA}	S_{QNA}
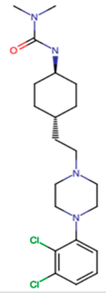	Cariprazine	0.843	0.732
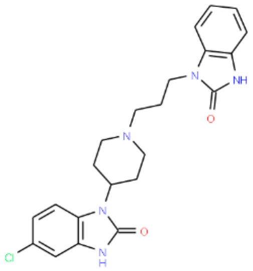	Domperidone	0.309	0.435
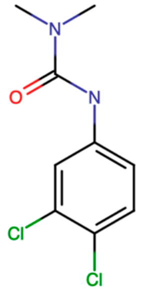	Diuron	0.404	0.415
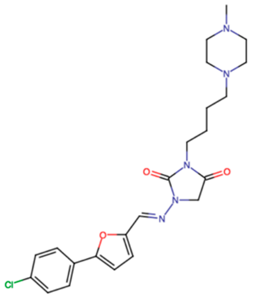	Azimilide	.value<0.3	0.339
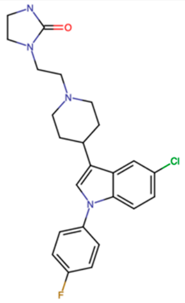	Sertindole	.value<0.3	0.337

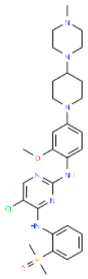	Brigatinib	.value<0.3	0.334
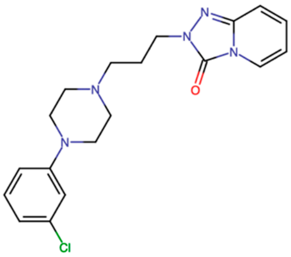	Trazodone	.value<0.3	0.328
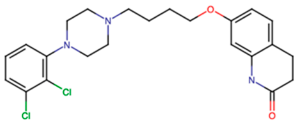	Aripiprazole	0.324	0.325
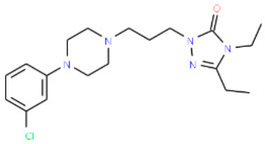	Etoperidone	.value<0.3	0.318
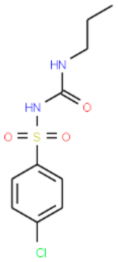	Chlorpropamide	.value<0.3	0.314
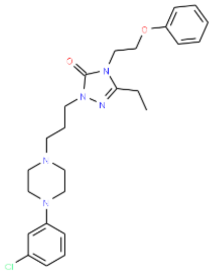	Nefazodone	.value<0.3	0.313

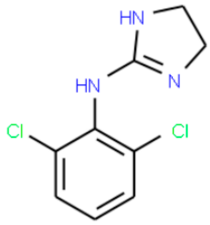	Clonidine	.value<0.3	0.310
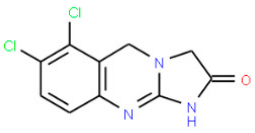	Anagrelide	.value<0.3	0.309
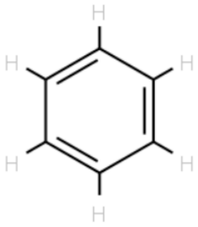	Benzene	.value<0.3	0.305
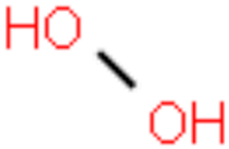	Hydrogen peroxide	.value<0.3	0.301
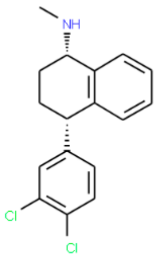	Sertraline	0.333	.value<0.3

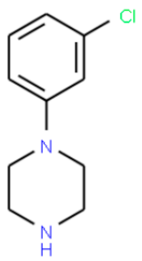	m-Chloropheylpiperazine	0.315	.value<0.3
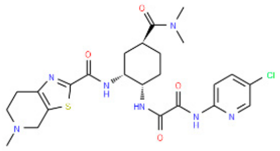	EDOXABAN	0.310	.value<0.3
